# Supplementary material for: Norms for 150 consumer products: Perceived complexity, quality objectivity, material/experiential nature, perceived price, familiarity and attitude
Source: PLoS One. 2020 Sep 21;15(9):e0238848. doi: 10.1371/journal.pone.0238848 (PMC7505470; doi:10.1371/journal.pone.0238848)
Supplement: S1 File — (DOCX) [file pone.0238848.s001.docx]

Supplemental Materials

Table 1. Descriptive statistics for product’s perceived complexity (ordered in descending order).

|  |  |  |  |  |  |  |  | **Items** | | |  | | |  |
| --- | --- | --- | --- | --- | --- | --- | --- | --- | --- | --- | --- | --- | --- | --- |
| **Product** | **Mean (across 3 items)** | **SD** | **CI -95** | **CI 95** | **Min** | **Max** | **1. How complex is this product?** | | **2. How many aspects of this product could you take into account when making a purchase decision?** | **3. To what extent is this product relatively unidimensional or relatively multidimensional?** | | **n** |  |  |
| **Car** | 5.12 | 1.06 | 4.85 | 5.39 | 2.33 | 6.00 | 5.13 | | 4.97 | 5.26 | | 61 |  |  |
| House | 5.12 | 0.95 | 4.67 | 5.56 | 3.00 | 6.00 | 4.85 | | 5.25 | 5.25 | | 20 |  |  |
| Hybrid/Convertible laptop | 5.10 | 0.80 | 4.73 | 5.47 | 3.67 | 6.00 | 5.05 | | 5.15 | 5.10 | | 20 |  |  |
| Laptop | 5.08 | 1.13 | 4.57 | 5.59 | 2.67 | 6.00 | 5.24 | | 4.95 | 5.05 | | 21 |  |  |
| Smartphone | 5.05 | 1.05 | 4.57 | 5.53 | 2.67 | 6.00 | 5.10 | | 5.05 | 5.00 | | 21 |  |  |
| **Desktop computer** | 4.98 | 0.96 | 4.73 | 5.22 | 2.33 | 6.00 | 5.07 | | 4.84 | 5.03 | | 61 |  |  |
| 3D printer | 4.84 | 1.30 | 4.25 | 5.43 | 2.33 | 6.00 | 5.00 | | 4.62 | 4.90 | | 21 |  |  |
| Motorcycle | 4.83 | 0.93 | 4.40 | 5.27 | 3.33 | 6.00 | 5.00 | | 4.70 | 4.80 | | 20 |  |  |
| Medical treatment | 4.81 | 1.28 | 4.23 | 5.39 | 2.00 | 6.00 | 5.05 | | 4.67 | 4.71 | | 21 |  |  |
| Life insurance | 4.49 | 1.18 | 3.96 | 5.03 | 2.33 | 6.00 | 4.90 | | 4.33 | 4.24 | | 21 |  |  |
| Vacation pack | 4.43 | 1.15 | 3.90 | 4.97 | 2.00 | 6.00 | 4.35 | | 4.60 | 4.35 | | 20 |  |  |
| Apartment | 4.40 | 1.26 | 3.82 | 4.97 | 2.33 | 6.00 | 4.38 | | 4.48 | 4.33 | | 21 |  |  |
| Tablet | 4.37 | 1.41 | 3.72 | 5.01 | 1.00 | 6.00 | 4.38 | | 4.19 | 4.52 | | 21 |  |  |
| Camera | 4.33 | 0.97 | 3.89 | 4.77 | 2.67 | 6.00 | 4.52 | | 4.10 | 4.38 | | 21 |  |  |
| Piano | 4.32 | 0.95 | 3.87 | 4.76 | 2.67 | 6.00 | 4.55 | | 4.15 | 4.25 | | 20 |  |  |
| **Room (renting)** | 4.26 | 1.19 | 3.96 | 4.57 | 2.00 | 6.00 | 4.05 | | 4.36 | 4.38 | | 61 |  |  |
| Cruise trip | 4.20 | 1.13 | 3.67 | 4.73 | 2.67 | 6.00 | 4.10 | | 4.15 | 4.35 | | 20 |  |  |
| Television | 4.15 | 1.11 | 3.63 | 4.67 | 2.00 | 6.00 | 4.10 | | 4.00 | 4.35 | | 20 |  |  |
| Printer | 3.97 | 1.30 | 3.36 | 4.58 | 1.33 | 6.00 | 4.35 | | 3.70 | 3.85 | | 20 |  |  |
| Internet provider | 3.95 | 1.27 | 3.37 | 4.53 | 1.67 | 6.00 | 4.05 | | 3.76 | 4.05 | | 21 |  |  |
| PC game | 3.95 | 1.03 | 3.47 | 4.43 | 2.00 | 6.00 | 4.05 | | 3.70 | 4.10 | | 20 |  |  |
| GPS | 3.95 | 1.00 | 3.48 | 4.42 | 1.00 | 6.00 | 4.30 | | 3.65 | 3.90 | | 20 |  |  |
| Speakers | 3.83 | 1.01 | 3.36 | 4.30 | 1.33 | 5.33 | 4.05 | | 3.55 | 3.90 | | 20 |  |  |
| Painting | 3.82 | 1.09 | 3.31 | 4.33 | 1.67 | 6.00 | 4.00 | | 3.60 | 3.85 | | 20 |  |  |
| Microwave | 3.82 | 1.13 | 3.29 | 4.35 | 1.33 | 5.33 | 3.95 | | 3.60 | 3.90 | | 20 |  |  |
| Hotel room | 3.78 | 1.20 | 3.23 | 4.33 | 2.00 | 5.33 | 3.90 | | 3.76 | 3.67 | | 21 |  |  |
| Air conditioner | 3.67 | 1.06 | 3.17 | 4.16 | 1.00 | 5.00 | 3.90 | | 3.45 | 3.65 | | 20 |  |  |
| Eyeglasses | 3.63 | 0.73 | 3.30 | 3.97 | 2.33 | 5.00 | 4.29 | | 3.43 | 3.19 | | 21 |  |  |
| Guitar | 3.63 | 1.38 | 2.99 | 4.28 | 1.00 | 6.00 | 3.65 | | 3.45 | 3.80 | | 20 |  |  |
| Gas outdoor grill | 3.60 | 0.82 | 3.22 | 3.98 | 2.00 | 5.33 | 4.00 | | 3.30 | 3.50 | | 20 |  |  |
| Watch | 3.53 | 1.30 | 2.92 | 4.14 | 1.00 | 6.00 | 3.85 | | 3.45 | 3.30 | | 20 |  |  |
| Dinner at a restaurant | 3.50 | 1.24 | 2.92 | 4.08 | 1.00 | 6.00 | 3.70 | | 3.10 | 3.70 | | 20 |  |  |
| Radio | 3.50 | 1.23 | 2.92 | 4.08 | 2.00 | 6.00 | 3.85 | | 3.10 | 3.55 | | 20 |  |  |
| Mattress | 3.48 | 0.95 | 3.04 | 3.93 | 2.00 | 6.00 | 3.45 | | 3.50 | 3.50 | | 20 |  |  |
| Online streaming subscription | 3.45 | 1.21 | 2.88 | 4.02 | 1.00 | 5.00 | 3.55 | | 3.35 | 3.45 | | 20 |  |  |
| DVD player | 3.41 | 1.28 | 2.83 | 3.99 | 1.67 | 6.00 | 3.52 | | 3.33 | 3.38 | | 21 |  |  |
| Suit | 3.37 | 1.13 | 2.84 | 3.90 | 1.00 | 5.33 | 3.40 | | 3.50 | 3.20 | | 20 |  |  |
| Calculator | 3.33 | 1.38 | 2.69 | 3.98 | 1.00 | 5.67 | 3.50 | | 3.10 | 3.40 | | 20 |  |  |
| Vacuum cleaner | 3.32 | 0.77 | 2.97 | 3.67 | 1.67 | 5.00 | 3.57 | | 3.33 | 3.05 | | 21 |  |  |
| Bicycle | 3.32 | 1.09 | 2.80 | 3.83 | 1.33 | 5.00 | 3.30 | | 3.25 | 3.40 | | 20 |  |  |
| Contact lenses | 3.22 | 1.11 | 2.70 | 3.74 | 1.00 | 5.33 | 3.40 | | 3.10 | 3.15 | | 20 |  |  |
| Gym subscription | 3.21 | 0.81 | 2.84 | 3.57 | 2.00 | 5.00 | 3.19 | | 3.14 | 3.29 | | 21 |  |  |
| Desk | 3.13 | 0.92 | 2.71 | 3.55 | 1.33 | 4.67 | 3.10 | | 3.19 | 3.10 | | 21 |  |  |
| Plane ticket | 3.11 | 1.28 | 2.53 | 3.69 | 1.00 | 5.00 | 3.05 | | 3.19 | 3.10 | | 21 |  |  |
| Power bank | 3.11 | 0.69 | 2.80 | 3.43 | 1.67 | 4.33 | 3.29 | | 2.81 | 3.19 | | 21 |  |  |
| Headphones | 3.10 | 1.02 | 2.62 | 3.58 | 1.67 | 5.00 | 3.15 | | 3.00 | 3.15 | | 20 |  |  |
| Wine | 3.08 | 1.08 | 2.58 | 3.59 | 1.33 | 5.00 | 3.30 | | 3.00 | 2.95 | | 20 |  |  |
| Couch | 3.07 | 0.73 | 2.72 | 3.41 | 2.00 | 5.00 | 3.10 | | 3.20 | 2.90 | | 20 |  |  |
| USB flash drive | 3.05 | 1.13 | 2.52 | 3.58 | 1.00 | 5.00 | 3.50 | | 2.60 | 3.05 | | 20 |  |  |
| Perfume | 3.03 | 1.18 | 2.48 | 3.59 | 1.00 | 6.00 | 3.35 | | 2.70 | 3.05 | | 20 |  |  |
| Book | 3.02 | 0.79 | 2.66 | 3.37 | 1.67 | 4.33 | 3.24 | | 2.71 | 3.10 | | 21 |  |  |
| Backpack | 2.95 | 0.86 | 2.56 | 3.35 | 2.00 | 4.67 | 2.90 | | 2.90 | 3.05 | | 21 |  |  |
| Toaster | 2.95 | 1.29 | 2.35 | 3.55 | 1.00 | 5.00 | 3.15 | | 2.75 | 2.95 | | 20 |  |  |
| Shoes | 2.92 | 0.98 | 2.46 | 3.37 | 2.00 | 5.00 | 3.05 | | 2.85 | 2.85 | | 20 |  |  |
| Makeup | 2.90 | 1.28 | 2.30 | 3.50 | 1.00 | 6.00 | 3.00 | | 2.90 | 2.80 | | 20 |  |  |
| Bracelet | 2.86 | 0.92 | 2.44 | 3.28 | 1.33 | 5.00 | 3.24 | | 2.86 | 2.48 | | 21 |  |  |
| Entree | 2.80 | 1.28 | 2.20 | 3.40 | 1.00 | 5.33 | 2.80 | | 2.70 | 2.90 | | 20 |  |  |
| Pants | 2.78 | 1.00 | 2.31 | 3.25 | 1.00 | 5.00 | 2.75 | | 2.85 | 2.75 | | 20 |  |  |
| Insecticide | 2.76 | 1.03 | 2.29 | 3.23 | 1.00 | 5.00 | 3.00 | | 2.71 | 2.57 | | 21 |  |  |
| Laptop bag | 2.75 | 1.24 | 2.17 | 3.33 | 1.00 | 6.00 | 2.55 | | 2.95 | 2.75 | | 20 |  |  |
| Massage | 2.73 | 1.07 | 2.23 | 3.24 | 1.00 | 6.00 | 2.80 | | 2.70 | 2.70 | | 20 |  |  |
| Shampoo | 2.73 | 1.20 | 2.17 | 3.29 | 1.00 | 4.67 | 2.70 | | 2.70 | 2.80 | | 20 |  |  |
| Pizza | 2.70 | 0.93 | 2.27 | 3.12 | 1.00 | 4.67 | 2.81 | | 2.76 | 2.52 | | 21 |  |  |
| Beer | 2.68 | 1.32 | 2.07 | 3.30 | 1.00 | 5.00 | 2.70 | | 2.60 | 2.75 | | 20 |  |  |
| Dress | 2.68 | 0.74 | 2.35 | 3.02 | 1.67 | 4.33 | 2.86 | | 2.57 | 2.57 | | 21 |  |  |
| CD | 2.68 | 0.96 | 2.25 | 3.12 | 1.33 | 4.00 | 2.57 | | 2.86 | 2.62 | | 21 |  |  |
| Sunscreen | 2.65 | 0.92 | 2.23 | 3.07 | 1.00 | 4.33 | 2.86 | | 2.52 | 2.57 | | 21 |  |  |
| DVD | 2.65 | 1.30 | 2.04 | 3.26 | 1.00 | 6.00 | 2.80 | | 2.40 | 2.75 | | 20 |  |  |
| Skin | 2.63 | 1.25 | 2.05 | 3.22 | 1.00 | 5.00 | 2.65 | | 2.60 | 2.65 | | 20 |  |  |
| Computer mouse | 2.62 | 0.93 | 2.18 | 3.05 | 1.00 | 4.00 | 2.70 | | 2.40 | 2.75 | | 20 |  |  |
| Table | 2.60 | 0.85 | 2.22 | 2.99 | 1.00 | 4.33 | 2.71 | | 2.67 | 2.43 | | 21 |  |  |
| Boots | 2.60 | 0.99 | 2.13 | 3.07 | 1.00 | 5.00 | 2.65 | | 2.55 | 2.60 | | 20 |  |  |
| Bedding | 2.57 | 0.88 | 2.17 | 2.97 | 1.00 | 4.33 | 2.52 | | 2.76 | 2.43 | | 21 |  |  |
| Bread | 2.57 | 1.12 | 2.04 | 3.09 | 1.00 | 5.00 | 2.65 | | 2.50 | 2.55 | | 20 |  |  |
| Vegetables | 2.55 | 1.36 | 1.91 | 3.19 | 1.00 | 5.33 | 2.45 | | 2.65 | 2.55 | | 20 |  |  |
| Shirt | 2.54 | 0.97 | 2.10 | 2.98 | 1.00 | 4.00 | 2.62 | | 2.57 | 2.43 | | 21 |  |  |
| Whiskey | 2.52 | 0.99 | 2.05 | 2.98 | 1.00 | 4.67 | 2.60 | | 2.35 | 2.60 | | 20 |  |  |
| Coffee | 2.51 | 0.98 | 2.06 | 2.95 | 1.00 | 4.33 | 2.67 | | 2.52 | 2.33 | | 21 |  |  |
| Plants | 2.49 | 0.90 | 2.08 | 2.90 | 1.00 | 4.33 | 2.52 | | 2.48 | 2.48 | | 21 |  |  |
| Cookies | 2.47 | 1.31 | 1.85 | 3.08 | 1.00 | 5.00 | 2.55 | | 2.35 | 2.50 | | 20 |  |  |
| Ice cream | 2.47 | 1.26 | 1.88 | 3.06 | 1.00 | 5.00 | 2.65 | | 2.35 | 2.40 | | 20 |  |  |
| Aspirin | 2.43 | 1.48 | 1.74 | 3.12 | 1.00 | 5.33 | 2.65 | | 2.30 | 2.35 | | 20 |  |  |
| Necklace | 2.43 | 0.89 | 2.02 | 2.85 | 1.00 | 5.00 | 2.50 | | 2.45 | 2.35 | | 20 |  |  |
| Wall clock | 2.43 | 0.87 | 2.03 | 2.84 | 1.00 | 4.33 | 2.45 | | 2.30 | 2.55 | | 20 |  |  |
| Museum ticket | 2.42 | 1.32 | 1.80 | 3.03 | 1.00 | 5.00 | 2.45 | | 2.45 | 2.35 | | 20 |  |  |
| Pillow | 2.40 | 0.90 | 1.98 | 2.82 | 1.00 | 3.67 | 2.45 | | 2.40 | 2.35 | | 20 |  |  |
| Chocolate | 2.40 | 1.27 | 1.80 | 3.00 | 1.00 | 5.00 | 2.50 | | 2.40 | 2.30 | | 20 |  |  |
| Chair | 2.40 | 0.82 | 2.02 | 2.78 | 1.00 | 3.67 | 2.35 | | 2.35 | 2.50 | | 20 |  |  |
| Nature park ticket | 2.38 | 0.89 | 1.97 | 2.80 | 1.00 | 4.00 | 2.20 | | 2.30 | 2.65 | | 20 |  |  |
| Appetizers | 2.38 | 1.07 | 1.88 | 2.89 | 1.00 | 5.67 | 2.50 | | 2.25 | 2.40 | | 20 |  |  |
| Concert ticket | 2.37 | 1.16 | 1.82 | 2.91 | 1.00 | 5.00 | 2.30 | | 2.45 | 2.35 | | 20 |  |  |
| Fairground | 2.37 | 1.21 | 1.80 | 2.93 | 1.00 | 5.00 | 2.35 | | 2.35 | 2.40 | | 20 |  |  |
| Blinds | 2.35 | 0.91 | 1.94 | 2.76 | 1.00 | 4.00 | 2.57 | | 2.29 | 2.19 | | 21 |  |  |
| Yogurt | 2.30 | 0.80 | 1.94 | 2.66 | 1.33 | 4.33 | 2.48 | | 2.24 | 2.19 | | 21 |  |  |
| Flashlight | 2.30 | 1.02 | 1.82 | 2.78 | 1.00 | 5.67 | 2.30 | | 2.30 | 2.30 | | 20 |  |  |
| Cutlery | 2.29 | 0.63 | 2.00 | 2.57 | 1.00 | 3.67 | 2.52 | | 2.10 | 2.24 | | 21 |  |  |
| Underwear | 2.28 | 1.18 | 1.73 | 2.84 | 1.00 | 4.67 | 2.25 | | 2.45 | 2.15 | | 20 |  |  |
| After shave | 2.28 | 1.06 | 1.79 | 2.78 | 1.00 | 4.33 | 2.40 | | 2.25 | 2.20 | | 20 |  |  |
| Chewing | 2.27 | 1.47 | 1.58 | 2.95 | 1.00 | 5.67 | 2.40 | | 2.30 | 2.10 | | 20 |  |  |
| Tea | 2.25 | 0.82 | 1.88 | 2.63 | 1.00 | 4.67 | 2.33 | | 2.33 | 2.10 | | 21 |  |  |
| Mouthwash | 2.25 | 1.00 | 1.78 | 2.72 | 1.00 | 5.00 | 2.30 | | 2.15 | 2.30 | | 20 |  |  |
| Shower gel | 2.22 | 0.73 | 1.89 | 2.56 | 1.00 | 3.67 | 2.48 | | 2.14 | 2.05 | | 21 |  |  |
| Pen | 2.22 | 0.85 | 1.82 | 2.61 | 1.00 | 4.33 | 2.20 | | 2.35 | 2.10 | | 20 |  |  |
| Soap | 2.19 | 1.11 | 1.68 | 2.70 | 1.00 | 5.00 | 2.29 | | 2.19 | 2.10 | | 21 |  |  |
| **Umbrella** | 2.19 | 0.70 | 2.01 | 2.37 | 1.00 | 4.00 | 2.16 | | 2.13 | 2.26 | | 61 |  |  |
| Dish detergent | 2.17 | 0.78 | 1.82 | 2.53 | 1.00 | 3.67 | 2.24 | | 2.38 | 1.90 | | 21 |  |  |
| Movie ticket | 2.17 | 1.22 | 1.60 | 2.74 | 1.00 | 6.00 | 2.10 | | 2.10 | 2.30 | | 20 |  |  |
| Laundry detergent | 2.15 | 0.85 | 1.75 | 2.55 | 1.00 | 4.00 | 2.25 | | 2.15 | 2.05 | | 20 |  |  |
| Baby diapers | 2.14 | 0.65 | 1.84 | 2.44 | 1.00 | 3.67 | 2.14 | | 2.10 | 2.14 | | 21 |  |  |
| Dessert at a restaurant | 2.13 | 0.53 | 1.88 | 2.37 | 1.33 | 3.67 | 2.43 | | 2.10 | 1.86 | | 21 |  |  |
| Fruit | 2.13 | 0.95 | 1.70 | 2.56 | 1.00 | 4.67 | 2.19 | | 2.05 | 2.14 | | 21 |  |  |
| Sun glasses | 2.12 | 0.62 | 1.82 | 2.41 | 1.00 | 3.33 | 1.90 | | 2.35 | 2.10 | | 20 |  |  |
| T-shirt | 2.12 | 1.15 | 1.58 | 2.65 | 1.00 | 6.00 | 2.05 | | 2.15 | 2.15 | | 20 |  |  |
| Water bottle | 2.11 | 0.83 | 1.73 | 2.49 | 1.00 | 3.67 | 2.14 | | 1.95 | 2.24 | | 21 |  |  |
| Peanut butter | 2.11 | 1.02 | 1.65 | 2.57 | 1.00 | 4.00 | 2.10 | | 2.19 | 2.05 | | 21 |  |  |
| Glasses (drinking) | 2.08 | 0.82 | 1.70 | 2.47 | 1.00 | 3.33 | 2.15 | | 2.10 | 2.00 | | 20 |  |  |
| Milk | 2.08 | 0.91 | 1.66 | 2.51 | 1.00 | 3.67 | 2.15 | | 2.05 | 2.05 | | 20 |  |  |
| Ceramic mug | 2.08 | 0.74 | 1.74 | 2.42 | 1.00 | 3.67 | 2.14 | | 2.00 | 2.10 | | 21 |  |  |
| Curtains | 2.07 | 0.82 | 1.68 | 2.45 | 1.00 | 3.33 | 2.25 | | 2.05 | 1.90 | | 20 |  |  |
| Vase | 2.07 | 0.98 | 1.61 | 2.52 | 1.00 | 4.67 | 2.20 | | 1.95 | 2.05 | | 20 |  |  |
| Soft drink | 2.03 | 1.24 | 1.45 | 2.61 | 1.00 | 6.00 | 2.00 | | 2.15 | 1.95 | | 20 |  |  |
| Air freshener | 2.02 | 0.80 | 1.64 | 2.39 | 1.00 | 3.67 | 2.10 | | 2.05 | 1.90 | | 20 |  |  |
| Breakfast cereal | 2.02 | 0.72 | 1.68 | 2.35 | 1.00 | 3.67 | 2.05 | | 2.25 | 1.75 | | 20 |  |  |
| Cooking oil | 2.02 | 1.33 | 1.40 | 2.64 | 1.00 | 5.67 | 1.80 | | 1.95 | 2.30 | | 20 |  |  |
| Toothpaste | 2.00 | 1.09 | 1.49 | 2.51 | 1.00 | 6.00 | 1.95 | | 2.20 | 1.85 | | 20 |  |  |
| Adhesive bandage | 2.00 | 0.82 | 1.62 | 2.38 | 1.00 | 3.67 | 2.05 | | 2.00 | 1.95 | | 20 |  |  |
| Cigarettes | 1.97 | 0.89 | 1.56 | 2.38 | 1.00 | 3.67 | 2.05 | | 1.86 | 2.00 | | 21 |  |  |
| Trash bin | 1.97 | 0.92 | 1.53 | 2.40 | 1.00 | 4.33 | 1.90 | | 2.00 | 2.00 | | 20 |  |  |
| Picture Frame | 1.95 | 0.93 | 1.51 | 2.39 | 1.00 | 4.67 | 2.00 | | 2.15 | 1.70 | | 20 |  |  |
| Blanket | 1.95 | 0.70 | 1.62 | 2.28 | 1.00 | 3.67 | 1.95 | | 2.10 | 1.80 | | 20 |  |  |
| Deodorant | 1.93 | 0.90 | 1.51 | 2.35 | 1.00 | 4.00 | 1.90 | | 2.15 | 1.75 | | 20 |  |  |
| Disposable plastic cups | 1.92 | 1.06 | 1.44 | 2.40 | 1.00 | 4.67 | 1.86 | | 2.05 | 1.86 | | 21 |  |  |
| Toilet | 1.92 | 1.01 | 1.44 | 2.39 | 1.00 | 5.00 | 1.85 | | 2.15 | 1.75 | | 20 |  |  |
| Dental floss | 1.90 | 1.05 | 1.41 | 2.39 | 1.00 | 4.67 | 1.95 | | 1.85 | 1.90 | | 20 |  |  |
| Scissors | 1.88 | 0.74 | 1.54 | 2.23 | 1.00 | 3.67 | 1.95 | | 1.90 | 1.80 | | 20 |  |  |
| Notebook | 1.86 | 0.77 | 1.51 | 2.21 | 1.00 | 4.33 | 1.71 | | 1.86 | 2.00 | | 21 |  |  |
| Pajamas | 1.85 | 0.69 | 1.53 | 2.17 | 1.00 | 3.67 | 1.70 | | 2.05 | 1.80 | | 20 |  |  |
| **Dishwashing brush** | 1.84 | 0.81 | 1.63 | 2.05 | 1.00 | 5.00 | 1.72 | | 1.90 | 1.90 | | 61 |  |  |
| Towel | 1.84 | 0.68 | 1.53 | 2.15 | 1.00 | 3.67 | 1.81 | | 1.95 | 1.76 | | 21 |  |  |
| Potato chips | 1.82 | 0.86 | 1.41 | 2.22 | 1.00 | 3.67 | 1.70 | | 2.00 | 1.75 | | 20 |  |  |
| Razor blades | 1.80 | 0.89 | 1.38 | 2.22 | 1.00 | 4.33 | 1.60 | | 2.00 | 1.80 | | 20 |  |  |
| Eggs | 1.75 | 0.98 | 1.30 | 2.19 | 1.00 | 4.67 | 1.86 | | 1.76 | 1.62 | | 21 |  |  |
| Postcard | 1.70 | 0.75 | 1.35 | 2.05 | 1.00 | 3.67 | 1.40 | | 1.90 | 1.80 | | 20 |  |  |
| **Oven mitts** | 1.70 | 0.76 | 1.50 | 1.89 | 1.00 | 4.00 | 1.52 | | 1.85 | 1.72 | | 61 |  |  |
| paper clips | 1.60 | 0.88 | 1.19 | 2.01 | 1.00 | 4.00 | 1.45 | | 1.65 | 1.70 | | 20 |  |  |
| Trash bags | 1.52 | 0.70 | 1.19 | 1.85 | 1.00 | 3.33 | 1.35 | | 1.80 | 1.40 | | 20 |  |  |
| Cleaning cloth | 1.45 | 0.72 | 1.11 | 1.79 | 1.00 | 3.33 | 1.45 | | 1.45 | 1.45 | | 20 |  |  |
| Coasters | 1.45 | 0.62 | 1.16 | 1.74 | 1.00 | 3.33 | 1.35 | | 1.65 | 1.35 | | 20 |  |  |
| Hangers | 1.43 | 0.73 | 1.09 | 1.78 | 1.00 | 3.67 | 1.40 | | 1.55 | 1.35 | | 20 |  |  |
| Toilet brush | 1.38 | 0.79 | 1.01 | 1.75 | 1.00 | 3.67 | 1.30 | | 1.45 | 1.40 | | 20 |  |  |

Table 2. Descriptive statistics for product’s perceived quality objectivity (ordered in descending order).

|  |  |  |  |  |  |  |  | **Items** | | |  | | |  |
| --- | --- | --- | --- | --- | --- | --- | --- | --- | --- | --- | --- | --- | --- | --- |
| **Product** | **Mean (across 3 items)** | **SD** | **CI -95** | **CI 95** | **Min** | **Max** | **1. To what extent is the evaluation of this product’s qualities a subjective or an objective matter?** | | **2. To what extent does the quality of this product depend on a personal taste or is objectively the same for everyone?** | **3. To what extent is the quality of this product a matter of opinion or a function of facts?** | | **n** |  |  |
| Trash bin | 4.98 | 1.04 | 4.50 | 5.47 | 2.33 | 6.00 | 4.90 | | 4.85 | 5.20 | | 20 |  |  |
| Aspirin | 4.97 | 0.99 | 4.50 | 5.43 | 3.00 | 6.00 | 4.60 | | 5.15 | 5.15 | | 20 |  |  |
| Trash bag | 4.92 | 1.14 | 4.38 | 5.45 | 1.33 | 6.00 | 5.00 | | 4.85 | 4.90 | | 20 |  |  |
| Scissors | 4.92 | 0.92 | 4.49 | 5.35 | 3.33 | 6.00 | 4.80 | | 4.90 | 5.05 | | 20 |  |  |
| DVD player | 4.82 | 1.04 | 4.33 | 5.30 | 3.00 | 6.00 | 4.80 | | 4.80 | 4.85 | | 20 |  |  |
| Cleaning cloth | 4.81 | 1.04 | 4.34 | 5.28 | 3.33 | 6.00 | 4.43 | | 4.90 | 5.10 | | 21 |  |  |
| Calculator | 4.80 | 1.13 | 4.27 | 5.33 | 3.00 | 6.00 | 4.80 | | 4.75 | 4.85 | | 20 |  |  |
| **Paper clips** | 4.80 | 0.91 | 4.57 | 5.03 | 2.67 | 6.00 | 4.72 | | 4.80 | 4.87 | | 61 |  |  |
| Dishwashing brush | 4.75 | 0.99 | 4.29 | 5.20 | 3.00 | 6.00 | 4.86 | | 4.81 | 4.57 | | 21 |  |  |
| Insecticide | 4.70 | 1.05 | 4.21 | 5.19 | 3.00 | 6.00 | 4.65 | | 4.85 | 4.60 | | 20 |  |  |
| Radio | 4.68 | 0.90 | 4.26 | 5.11 | 3.00 | 6.00 | 4.65 | | 4.55 | 4.85 | | 20 |  |  |
| Disposable plastic cups | 4.65 | 0.99 | 4.18 | 5.12 | 2.00 | 6.00 | 4.60 | | 4.45 | 4.90 | | 20 |  |  |
| Adhesive bandage | 4.63 | 0.95 | 4.20 | 5.07 | 3.00 | 6.00 | 4.62 | | 4.57 | 4.71 | | 21 |  |  |
| USB flash drive | 4.63 | 1.12 | 4.11 | 5.16 | 2.67 | 6.00 | 4.60 | | 4.60 | 4.70 | | 20 |  |  |
| Toaster | 4.62 | 1.00 | 4.16 | 5.07 | 2.67 | 6.00 | 4.57 | | 4.52 | 4.76 | | 21 |  |  |
| Baby diapers | 4.60 | 0.73 | 4.26 | 4.94 | 3.67 | 6.00 | 4.45 | | 4.50 | 4.85 | | 20 |  |  |
| Internet provider | 4.60 | 1.06 | 4.10 | 5.10 | 3.00 | 6.00 | 4.75 | | 4.45 | 4.60 | | 20 |  |  |
| Flashlight | 4.60 | 1.01 | 4.13 | 5.07 | 2.67 | 6.00 | 4.50 | | 4.60 | 4.70 | | 20 |  |  |
| Printer | 4.60 | 1.12 | 4.07 | 5.13 | 2.33 | 6.00 | 4.35 | | 4.65 | 4.80 | | 20 |  |  |
| Laundry detergent | 4.57 | 0.88 | 4.17 | 4.97 | 3.33 | 6.00 | 4.43 | | 4.67 | 4.62 | | 21 |  |  |
| Toilet brush | 4.57 | 1.27 | 3.97 | 5.16 | 2.67 | 6.00 | 4.60 | | 4.50 | 4.60 | | 20 |  |  |
| Plane ticket | 4.53 | 1.11 | 4.02 | 5.05 | 2.00 | 6.00 | 4.55 | | 4.40 | 4.65 | | 20 |  |  |
| Notebook | 4.52 | 1.17 | 3.97 | 5.07 | 2.67 | 6.00 | 4.50 | | 4.50 | 4.55 | | 20 |  |  |
| GPS | 4.51 | 1.27 | 3.93 | 5.08 | 1.00 | 6.00 | 4.19 | | 4.52 | 4.81 | | 21 |  |  |
| Air conditioner | 4.49 | 1.12 | 3.98 | 5.00 | 2.00 | 6.00 | 4.48 | | 4.43 | 4.57 | | 21 |  |  |
| Dental floss | 4.48 | 1.27 | 3.89 | 5.08 | 1.33 | 6.00 | 4.45 | | 4.40 | 4.60 | | 20 |  |  |
| **Hangers** | 4.48 | 1.17 | 4.18 | 4.78 | 1.33 | 6.00 | 4.41 | | 4.48 | 4.56 | | 61 |  |  |
| Life insurance | 4.44 | 1.05 | 3.97 | 4.92 | 2.67 | 6.00 | 4.38 | | 4.43 | 4.52 | | 21 |  |  |
| 3D printer | 4.43 | 1.40 | 3.79 | 5.07 | 1.00 | 6.00 | 4.43 | | 4.33 | 4.52 | | 21 |  |  |
| Microwave | 4.42 | 0.88 | 4.00 | 4.83 | 2.33 | 6.00 | 4.30 | | 4.50 | 4.45 | | 20 |  |  |
| Speakers | 4.41 | 0.97 | 3.97 | 4.85 | 2.67 | 6.00 | 4.00 | | 4.48 | 4.76 | | 21 |  |  |
| Sunscreen | 4.41 | 1.22 | 3.86 | 4.97 | 1.00 | 6.00 | 4.33 | | 4.52 | 4.38 | | 21 |  |  |
| Razor blades | 4.33 | 1.29 | 3.73 | 4.94 | 1.00 | 6.00 | 4.40 | | 4.30 | 4.30 | | 20 |  |  |
| Vacuum cleaner | 4.33 | 1.24 | 3.75 | 4.91 | 1.67 | 6.00 | 4.35 | | 4.30 | 4.35 | | 20 |  |  |
| Umbrella | 4.33 | 1.32 | 3.72 | 4.95 | 1.00 | 6.00 | 4.10 | | 4.50 | 4.40 | | 20 |  |  |
| Television | 4.32 | 1.18 | 3.77 | 4.87 | 2.33 | 6.00 | 4.35 | | 4.05 | 4.55 | | 20 |  |  |
| Dish detergent | 4.30 | 1.15 | 3.78 | 4.82 | 1.00 | 6.00 | 4.38 | | 4.24 | 4.29 | | 21 |  |  |
| Cutlery | 4.29 | 1.04 | 3.81 | 4.76 | 2.67 | 6.00 | 4.19 | | 4.05 | 4.62 | | 21 |  |  |
| **Medical treatment** | 4.27 | 1.20 | 3.97 | 4.58 | 1.00 | 6.00 | 4.07 | | 4.25 | 4.51 | | 61 |  |  |
| Power bank | 4.25 | 1.17 | 3.70 | 4.80 | 1.67 | 6.00 | 4.10 | | 4.50 | 4.15 | | 20 |  |  |
| Eyeglasses | 4.24 | 1.13 | 3.72 | 4.75 | 2.00 | 6.00 | 4.38 | | 3.90 | 4.43 | | 21 |  |  |
| Piano | 4.24 | 1.22 | 3.68 | 4.79 | 1.00 | 6.00 | 4.19 | | 4.19 | 4.33 | | 21 |  |  |
| Laptop | 4.18 | 0.86 | 3.78 | 4.59 | 2.67 | 6.00 | 4.20 | | 4.20 | 4.15 | | 20 |  |  |
| Gas outdoor grill | 4.16 | 1.17 | 3.63 | 4.69 | 1.00 | 6.00 | 4.14 | | 3.90 | 4.43 | | 21 |  |  |
| Desktop computer | 4.16 | 1.38 | 3.53 | 4.79 | 1.00 | 6.00 | 4.10 | | 3.86 | 4.52 | | 21 |  |  |
| Water bottle | 4.15 | 1.05 | 3.66 | 4.64 | 2.00 | 6.00 | 4.05 | | 4.20 | 4.20 | | 20 |  |  |
| Toilet paper | 4.14 | 1.03 | 3.68 | 4.61 | 1.00 | 6.00 | 3.95 | | 4.05 | 4.43 | | 21 |  |  |
| Table | 4.13 | 1.19 | 3.58 | 4.69 | 2.33 | 6.00 | 4.45 | | 3.95 | 4.00 | | 20 |  |  |
| Soap | 4.10 | 0.80 | 3.73 | 4.46 | 3.00 | 6.00 | 4.10 | | 3.95 | 4.24 | | 21 |  |  |
| Coasters | 4.07 | 1.02 | 3.59 | 4.55 | 1.00 | 6.00 | 3.95 | | 4.00 | 4.25 | | 20 |  |  |
| Oven mitts | 4.05 | 1.26 | 3.46 | 4.64 | 1.67 | 6.00 | 3.90 | | 4.05 | 4.20 | | 20 |  |  |
| Computer mouse | 4.03 | 1.24 | 3.45 | 4.61 | 2.00 | 6.00 | 4.00 | | 3.80 | 4.30 | | 20 |  |  |
| Eggs | 4.02 | 1.22 | 3.46 | 4.57 | 2.00 | 6.00 | 4.14 | | 3.90 | 4.00 | | 21 |  |  |
| Towel | 4.00 | 1.10 | 3.50 | 4.50 | 1.00 | 6.00 | 3.95 | | 4.19 | 3.86 | | 21 |  |  |
| Contact lenses | 3.95 | 1.34 | 3.34 | 4.56 | 1.00 | 6.00 | 4.10 | | 3.76 | 4.00 | | 21 |  |  |
| Camera | 3.95 | 1.18 | 3.40 | 4.50 | 2.00 | 6.00 | 4.00 | | 3.70 | 4.15 | | 20 |  |  |
| Pen | 3.84 | 1.35 | 3.23 | 4.46 | 1.00 | 6.00 | 3.71 | | 3.86 | 3.95 | | 21 |  |  |
| Tablet | 3.83 | 1.29 | 3.23 | 4.44 | 1.67 | 6.00 | 3.60 | | 3.85 | 4.05 | | 20 |  |  |
| Picture frame | 3.83 | 1.44 | 3.16 | 4.51 | 1.00 | 6.00 | 3.90 | | 3.70 | 3.90 | | 20 |  |  |
| Hybrid/Convertible laptop | 3.78 | 1.43 | 3.11 | 4.45 | 1.00 | 6.00 | 3.60 | | 3.85 | 3.90 | | 20 |  |  |
| Guitar | 3.78 | 0.97 | 3.33 | 4.24 | 1.00 | 6.00 | 3.70 | | 3.55 | 4.10 | | 20 |  |  |
| Desk | 3.77 | 1.09 | 3.26 | 4.28 | 1.67 | 6.00 | 3.75 | | 3.85 | 3.70 | | 20 |  |  |
| Motorcycle | 3.77 | 1.01 | 3.29 | 4.24 | 2.00 | 6.00 | 3.80 | | 3.75 | 3.75 | | 20 |  |  |
| Laptop bag | 3.76 | 1.43 | 3.11 | 4.41 | 1.00 | 6.00 | 3.81 | | 3.86 | 3.62 | | 21 |  |  |
| Wall clock | 3.76 | 1.13 | 3.25 | 4.27 | 2.00 | 6.00 | 3.67 | | 3.76 | 3.86 | | 21 |  |  |
| Bicycle | 3.73 | 1.40 | 3.08 | 4.39 | 1.33 | 6.00 | 3.80 | | 3.60 | 3.80 | | 20 |  |  |
| Blanket | 3.73 | 1.30 | 3.12 | 4.34 | 1.67 | 6.00 | 3.85 | | 3.55 | 3.80 | | 20 |  |  |
| Chair | 3.72 | 0.98 | 3.26 | 4.18 | 2.00 | 5.67 | 3.85 | | 3.30 | 4.00 | | 20 |  |  |
| Mouthwash | 3.68 | 1.02 | 3.20 | 4.16 | 1.00 | 5.67 | 3.70 | | 3.45 | 3.90 | | 20 |  |  |
| Skin moisturizer | 3.68 | 1.27 | 3.11 | 4.26 | 1.00 | 6.00 | 3.67 | | 3.52 | 3.86 | | 21 |  |  |
| Headphones | 3.67 | 1.34 | 3.04 | 4.29 | 1.33 | 6.00 | 3.75 | | 3.60 | 3.65 | | 20 |  |  |
| Glasses (drinking) | 3.65 | 1.18 | 3.10 | 4.20 | 1.00 | 6.00 | 3.40 | | 3.45 | 4.10 | | 20 |  |  |
| Backpack | 3.63 | 1.16 | 3.09 | 4.18 | 1.67 | 6.00 | 3.50 | | 3.35 | 4.05 | | 20 |  |  |
| Pants | 3.62 | 1.02 | 3.16 | 4.08 | 1.00 | 6.00 | 3.71 | | 3.33 | 3.81 | | 21 |  |  |
| Blinds | 3.59 | 1.12 | 3.08 | 4.10 | 1.00 | 6.00 | 3.67 | | 3.57 | 3.52 | | 21 |  |  |
| Couch | 3.56 | 0.91 | 3.14 | 3.97 | 1.00 | 5.00 | 3.81 | | 3.48 | 3.38 | | 21 |  |  |
| Ceramic mug | 3.55 | 0.92 | 3.12 | 3.98 | 2.33 | 6.00 | 3.30 | | 3.40 | 3.95 | | 20 |  |  |
| Boots | 3.52 | 0.87 | 3.11 | 3.92 | 1.67 | 5.00 | 3.55 | | 3.40 | 3.60 | | 20 |  |  |
| Apartment | 3.50 | 0.74 | 3.15 | 3.85 | 1.67 | 4.67 | 3.45 | | 3.35 | 3.70 | | 20 |  |  |
| Milk | 3.50 | 1.17 | 2.95 | 4.05 | 1.00 | 6.00 | 3.35 | | 3.55 | 3.60 | | 20 |  |  |
| Underwear | 3.48 | 1.18 | 2.93 | 4.03 | 2.00 | 6.00 | 3.35 | | 3.30 | 3.80 | | 20 |  |  |
| Toothpaste | 3.38 | 1.13 | 2.85 | 3.91 | 1.00 | 6.00 | 3.15 | | 3.25 | 3.75 | | 20 |  |  |
| Cooking oil | 3.37 | 1.25 | 2.78 | 3.95 | 1.00 | 6.00 | 3.20 | | 3.60 | 3.30 | | 20 |  |  |
| Vegetables | 3.35 | 1.11 | 2.84 | 3.85 | 1.00 | 5.33 | 3.52 | | 3.00 | 3.52 | | 21 |  |  |
| T-shirt | 3.33 | 1.05 | 2.86 | 3.81 | 1.00 | 5.33 | 3.43 | | 3.00 | 3.57 | | 21 |  |  |
| Mattress | 3.33 | 0.97 | 2.88 | 3.79 | 1.33 | 6.00 | 3.35 | | 3.15 | 3.50 | | 20 |  |  |
| Museum ticket | 3.32 | 1.46 | 2.63 | 4.00 | 1.00 | 6.00 | 3.35 | | 3.25 | 3.35 | | 20 |  |  |
| Smartphone | 3.32 | 1.24 | 2.74 | 3.90 | 1.00 | 6.00 | 3.45 | | 3.20 | 3.30 | | 20 |  |  |
| Cigarettes | 3.24 | 1.15 | 2.72 | 3.76 | 1.00 | 6.00 | 3.43 | | 3.00 | 3.29 | | 21 |  |  |
| Hotel room | 3.23 | 1.14 | 2.70 | 3.77 | 1.67 | 6.00 | 3.05 | | 3.30 | 3.35 | | 20 |  |  |
| Shoes | 3.22 | 0.94 | 2.79 | 3.65 | 1.00 | 5.00 | 3.19 | | 3.10 | 3.38 | | 21 |  |  |
| Nature park ticket | 3.22 | 1.69 | 2.43 | 4.01 | 1.00 | 6.00 | 3.10 | | 3.30 | 3.25 | | 20 |  |  |
| Gym subscription | 3.22 | 0.93 | 2.78 | 3.65 | 1.00 | 4.67 | 3.25 | | 3.00 | 3.40 | | 20 |  |  |
| Fruit | 3.18 | 0.79 | 2.81 | 3.55 | 2.00 | 5.33 | 3.15 | | 3.05 | 3.35 | | 20 |  |  |
| Deodorant | 3.17 | 1.08 | 2.68 | 3.67 | 1.00 | 5.00 | 2.90 | | 3.24 | 3.38 | | 21 |  |  |
| Peanut butter | 3.17 | 1.24 | 2.59 | 3.75 | 1.00 | 6.00 | 3.45 | | 2.85 | 3.20 | | 20 |  |  |
| Car | 3.15 | 1.37 | 2.51 | 3.79 | 1.33 | 6.00 | 3.15 | | 2.90 | 3.40 | | 20 |  |  |
| House | 3.15 | 1.43 | 2.48 | 3.82 | 1.00 | 6.00 | 3.25 | | 3.10 | 3.10 | | 20 |  |  |
| Shower gel | 3.15 | 1.24 | 2.57 | 3.73 | 1.00 | 6.00 | 3.05 | | 3.30 | 3.10 | | 20 |  |  |
| Curtains | 3.14 | 0.98 | 2.70 | 3.59 | 1.00 | 5.00 | 3.10 | | 3.38 | 2.95 | | 21 |  |  |
| Watch | 3.12 | 1.14 | 2.59 | 3.65 | 1.33 | 6.00 | 3.05 | | 3.00 | 3.30 | | 20 |  |  |
| Room (renting) | 3.12 | 1.13 | 2.59 | 3.64 | 1.00 | 5.00 | 3.05 | | 3.10 | 3.20 | | 20 |  |  |
| Bedding | 3.03 | 1.51 | 2.33 | 3.74 | 1.00 | 6.00 | 3.05 | | 3.05 | 3.00 | | 20 |  |  |
| Suit | 3.03 | 0.79 | 2.66 | 3.40 | 1.00 | 4.33 | 2.85 | | 2.95 | 3.30 | | 20 |  |  |
| Necklace | 3.03 | 1.05 | 2.54 | 3.52 | 1.00 | 5.33 | 3.05 | | 2.95 | 3.10 | | 20 |  |  |
| Vase | 3.03 | 1.34 | 2.42 | 3.64 | 1.00 | 5.00 | 3.10 | | 3.00 | 3.00 | | 21 |  |  |
| Pajamas | 3.02 | 1.68 | 2.23 | 3.80 | 1.00 | 6.00 | 2.95 | | 2.90 | 3.20 | | 20 |  |  |
| Coffee | 3.02 | 1.26 | 2.43 | 3.61 | 1.00 | 5.33 | 2.95 | | 3.00 | 3.10 | | 20 |  |  |
| Pillow | 3.00 | 0.94 | 2.57 | 3.43 | 1.00 | 4.67 | 2.95 | | 2.71 | 3.33 | | 21 |  |  |
| Online streaming subscription | 3.00 | 1.14 | 2.47 | 3.53 | 1.00 | 4.67 | 2.90 | | 2.90 | 3.20 | | 20 |  |  |
| Postcard | 2.98 | 1.61 | 2.23 | 3.74 | 1.00 | 6.00 | 2.95 | | 2.90 | 3.10 | | 20 |  |  |
| Cruise trip | 2.97 | 1.31 | 2.36 | 3.58 | 1.00 | 6.00 | 2.90 | | 2.80 | 3.20 | | 20 |  |  |
| Potato chips | 2.95 | 1.03 | 2.48 | 3.42 | 1.00 | 5.00 | 3.19 | | 2.76 | 2.90 | | 21 |  |  |
| Movie ticket | 2.95 | 1.33 | 2.33 | 3.57 | 1.00 | 6.00 | 2.70 | | 2.90 | 3.25 | | 20 |  |  |
| Shirt | 2.93 | 1.50 | 2.23 | 3.64 | 1.00 | 6.00 | 2.80 | | 3.00 | 3.00 | | 20 |  |  |
| Chewing gum | 2.88 | 1.24 | 2.30 | 3.46 | 1.00 | 5.00 | 2.85 | | 2.70 | 3.10 | | 20 |  |  |
| **Vacation package** | 2.87 | 1.17 | 2.57 | 3.17 | 1.00 | 5.33 | 2.95 | | 2.77 | 2.89 | | 61 |  |  |
| Soft drink | 2.86 | 0.93 | 2.43 | 3.28 | 1.00 | 4.67 | 2.95 | | 2.76 | 2.86 | | 21 |  |  |
| Pizza | 2.85 | 1.51 | 2.14 | 3.56 | 1.00 | 6.00 | 2.95 | | 2.70 | 2.90 | | 20 |  |  |
| Bracelet | 2.82 | 1.50 | 2.11 | 3.52 | 1.00 | 6.00 | 2.75 | | 2.75 | 2.95 | | 20 |  |  |
| Dress | 2.75 | 1.25 | 2.16 | 3.34 | 1.00 | 5.00 | 2.80 | | 2.55 | 2.90 | | 20 |  |  |
| Shampoo | 2.73 | 1.28 | 2.14 | 3.33 | 1.00 | 6.00 | 2.55 | | 2.65 | 3.00 | | 20 |  |  |
| PC game | 2.73 | 1.26 | 2.16 | 3.31 | 1.00 | 5.00 | 2.71 | | 2.62 | 2.86 | | 21 |  |  |
| Plants | 2.71 | 1.00 | 2.26 | 3.17 | 1.00 | 4.33 | 2.71 | | 2.71 | 2.71 | | 21 |  |  |
| Air freshener | 2.65 | 0.96 | 2.22 | 3.09 | 1.00 | 4.33 | 2.76 | | 2.48 | 2.71 | | 21 |  |  |
| DVD | 2.60 | 1.44 | 1.93 | 3.27 | 1.00 | 6.00 | 2.45 | | 2.60 | 2.75 | | 20 |  |  |
| Sun glasses | 2.60 | 0.81 | 2.22 | 2.98 | 1.00 | 4.00 | 2.85 | | 2.30 | 2.65 | | 20 |  |  |
| **Dessert at a restaurant** | 2.58 | 1.26 | 2.26 | 2.91 | 1.00 | 5.00 | 2.54 | | 2.57 | 2.64 | | 61 |  |  |
| CD | 2.57 | 1.63 | 1.83 | 3.31 | 1.00 | 6.00 | 2.62 | | 2.52 | 2.57 | | 21 |  |  |
| After shave | 2.53 | 1.16 | 1.99 | 3.07 | 1.00 | 5.33 | 2.85 | | 2.25 | 2.50 | | 20 |  |  |
| Beer | 2.52 | 1.05 | 2.05 | 3.00 | 1.00 | 4.33 | 2.62 | | 2.24 | 2.71 | | 21 |  |  |
| Cookies | 2.50 | 1.08 | 1.99 | 3.01 | 1.00 | 4.33 | 2.50 | | 2.45 | 2.55 | | 20 |  |  |
| **Entrée at a restaurant** | 2.50 | 1.21 | 2.19 | 2.81 | 1.00 | 5.00 | 2.64 | | 2.36 | 2.49 | | 61 |  |  |
| Fairground ticket | 2.49 | 1.30 | 1.90 | 3.08 | 1.00 | 5.00 | 2.67 | | 2.48 | 2.33 | | 21 |  |  |
| Dinner at a restaurant | 2.48 | 1.23 | 1.91 | 3.06 | 1.00 | 4.67 | 2.55 | | 2.20 | 2.70 | | 20 |  |  |
| Massage | 2.42 | 1.17 | 1.87 | 2.96 | 1.00 | 4.67 | 2.50 | | 2.25 | 2.50 | | 20 |  |  |
| Painting | 2.41 | 1.17 | 1.88 | 2.95 | 1.00 | 4.67 | 2.38 | | 2.38 | 2.48 | | 21 |  |  |
| Bread | 2.40 | 1.11 | 1.88 | 2.92 | 1.00 | 4.67 | 2.75 | | 2.25 | 2.20 | | 20 |  |  |
| Wine | 2.40 | 1.17 | 1.86 | 2.93 | 1.00 | 4.67 | 2.67 | | 2.14 | 2.38 | | 21 |  |  |
| Perfume | 2.38 | 1.40 | 1.73 | 3.04 | 1.00 | 5.00 | 2.55 | | 2.30 | 2.30 | | 20 |  |  |
| Book | 2.28 | 1.22 | 1.71 | 2.85 | 1.00 | 5.00 | 2.25 | | 2.20 | 2.40 | | 20 |  |  |
| Concert ticket | 2.25 | 1.25 | 1.69 | 2.82 | 1.00 | 4.67 | 2.33 | | 2.29 | 2.14 | | 21 |  |  |
| Yogurt | 2.17 | 0.87 | 1.76 | 2.57 | 1.00 | 4.33 | 2.40 | | 2.10 | 2.00 | | 20 |  |  |
| Appetizers | 2.05 | 0.98 | 1.59 | 2.51 | 1.00 | 4.33 | 2.20 | | 1.90 | 2.05 | | 20 |  |  |
| Makeup | 2.02 | 1.06 | 1.52 | 2.51 | 1.00 | 4.33 | 1.85 | | 1.95 | 2.25 | | 20 |  |  |
| Breakfast cereal | 2.02 | 0.89 | 1.60 | 2.43 | 1.00 | 3.67 | 2.00 | | 1.85 | 2.20 | | 20 |  |  |
| Ice cream | 1.97 | 0.76 | 1.61 | 2.32 | 1.00 | 3.67 | 1.95 | | 1.80 | 2.15 | | 20 |  |  |
| Whiskey | 1.97 | 0.76 | 1.61 | 2.32 | 1.00 | 3.33 | 1.95 | | 1.90 | 2.05 | | 20 |  |  |
| Tea | 1.97 | 0.82 | 1.58 | 2.35 | 1.00 | 3.67 | 2.10 | | 1.75 | 2.05 | | 20 |  |  |
| Chocolate bar | 1.80 | 0.93 | 1.37 | 2.23 | 1.00 | 4.67 | 1.75 | | 1.60 | 2.05 | | 20 |  |  |

Table 3. Descriptive statistics for product’s experiential/material nature (ordered in descending order).

|  |  |  |  |  |  |  |  | **Items** | | |  | | |  |
| --- | --- | --- | --- | --- | --- | --- | --- | --- | --- | --- | --- | --- | --- | --- |
| **Product** | **Mean (across 3 items)** | **SD** | **CI -95** | **CI 95** | **Min** | **Max** | **1. To what extent is the purchase of this product a material purchase or an experiential purchase?** | | **2. To what extent does the purchase of this product emphasize possession of an object or experiencing an activity?** | **3. To what extent is the purchase of this product focused on having or focused on doing?** | | **n** |  |  |
| Massage | 5.64 | 0.74 | 5.31 | 5.96 | 3.00 | 6.00 | 5.73 | | 5.77 | 5.41 | | 22 |  |  |
| Cruise trip | 5.56 | 0.65 | 5.26 | 5.85 | 4.00 | 6.00 | 5.48 | | 5.71 | 5.48 | | 21 |  |  |
| **Vacation package** | 5.40 | 0.86 | 5.18 | 5.61 | 2.33 | 6.00 | 5.31 | | 5.41 | 5.47 | | 64 |  |  |
| **Museum ticket** | 5.27 | 1.01 | 5.02 | 5.52 | 2.33 | 6.00 | 5.13 | | 5.36 | 5.33 | | 64 |  |  |
| Concert ticket | 5.22 | 1.53 | 4.53 | 5.92 | 1.00 | 6.00 | 5.19 | | 5.29 | 5.19 | | 21 |  |  |
| Fairground ticket | 5.10 | 1.16 | 4.56 | 5.63 | 2.67 | 6.00 | 4.95 | | 5.19 | 5.14 | | 21 |  |  |
| Hotel room | 5.03 | 1.23 | 4.48 | 5.58 | 1.00 | 6.00 | 5.00 | | 5.09 | 5.00 | | 22 |  |  |
| Movie ticket | 4.97 | 1.22 | 4.41 | 5.53 | 2.00 | 6.00 | 4.71 | | 5.10 | 5.10 | | 21 |  |  |
| Nature park ticket | 4.97 | 1.12 | 4.46 | 5.48 | 2.00 | 6.00 | 4.90 | | 5.00 | 5.00 | | 21 |  |  |
| Dessert at a restaurant | 4.88 | 1.36 | 4.28 | 5.48 | 1.00 | 6.00 | 4.73 | | 5.05 | 4.86 | | 22 |  |  |
| Gym subscription | 4.87 | 1.03 | 4.40 | 5.34 | 3.00 | 6.00 | 4.67 | | 5.05 | 4.90 | | 21 |  |  |
| Medical treatment | 4.79 | 1.15 | 4.27 | 5.32 | 2.33 | 6.00 | 4.86 | | 4.81 | 4.71 | | 21 |  |  |
| **Dinner at a restaurant** | 4.79 | 1.06 | 4.52 | 5.05 | 1.00 | 6.00 | 4.84 | | 4.80 | 4.72 | | 64 |  |  |
| Plane ticket | 4.71 | 1.51 | 4.03 | 5.40 | 1.00 | 6.00 | 4.71 | | 4.62 | 4.81 | | 21 |  |  |
| Online streaming subscription | 4.46 | 1.49 | 3.78 | 5.14 | 1.00 | 6.00 | 4.67 | | 4.33 | 4.38 | | 21 |  |  |
| Pizza | 4.42 | 1.34 | 3.83 | 5.02 | 1.67 | 6.00 | 4.36 | | 4.41 | 4.50 | | 22 |  |  |
| PC game | 4.37 | 1.24 | 3.80 | 4.93 | 1.00 | 6.00 | 4.14 | | 4.19 | 4.76 | | 21 |  |  |
| Internet provider | 4.29 | 1.62 | 3.55 | 5.02 | 1.00 | 6.00 | 4.19 | | 4.52 | 4.14 | | 21 |  |  |
| Appetizers | 4.11 | 1.54 | 3.41 | 4.81 | 1.00 | 6.00 | 3.86 | | 4.57 | 3.90 | | 21 |  |  |
| Wine | 4.03 | 1.32 | 3.43 | 4.63 | 1.00 | 6.00 | 3.81 | | 4.05 | 4.24 | | 21 |  |  |
| Entrée at a restaurant | 4.00 | 1.80 | 3.18 | 4.82 | 1.00 | 6.00 | 3.95 | | 4.24 | 3.81 | | 21 |  |  |
| Peanut butter | 3.95 | 1.59 | 3.23 | 4.67 | 1.00 | 6.00 | 3.76 | | 4.00 | 4.10 | | 21 |  |  |
| Beer | 3.92 | 1.48 | 3.25 | 4.59 | 1.00 | 6.00 | 3.43 | | 4.29 | 4.05 | | 21 |  |  |
| Soft drink | 3.87 | 1.38 | 3.25 | 4.50 | 2.00 | 6.00 | 3.76 | | 4.00 | 3.86 | | 21 |  |  |
| Chewing gum | 3.83 | 1.41 | 3.18 | 4.47 | 1.33 | 6.00 | 3.62 | | 3.71 | 4.14 | | 21 |  |  |
| DVD | 3.78 | 1.23 | 3.22 | 4.34 | 1.00 | 5.67 | 3.62 | | 3.90 | 3.81 | | 21 |  |  |
| Coffee | 3.75 | 1.62 | 3.01 | 4.48 | 1.00 | 6.00 | 3.67 | | 4.14 | 3.43 | | 21 |  |  |
| Cookies | 3.73 | 1.72 | 2.96 | 4.49 | 1.00 | 6.00 | 3.45 | | 3.82 | 3.91 | | 22 |  |  |
| Bicycle | 3.67 | 0.96 | 3.23 | 4.10 | 2.00 | 6.00 | 3.14 | | 3.43 | 4.43 | | 21 |  |  |
| Contact lenses | 3.67 | 1.63 | 2.93 | 4.41 | 1.00 | 6.00 | 3.52 | | 3.67 | 3.81 | | 21 |  |  |
| Fruit | 3.63 | 1.26 | 3.06 | 4.21 | 1.00 | 5.00 | 3.43 | | 3.71 | 3.76 | | 21 |  |  |
| Potato chips | 3.62 | 1.42 | 2.97 | 4.26 | 1.00 | 6.00 | 3.24 | | 4.00 | 3.62 | | 21 |  |  |
| Ice cream | 3.52 | 1.74 | 2.74 | 4.29 | 1.00 | 6.00 | 3.41 | | 3.68 | 3.45 | | 22 |  |  |
| Chocolate bar | 3.51 | 1.85 | 2.67 | 4.35 | 1.00 | 6.00 | 3.24 | | 3.76 | 3.52 | | 21 |  |  |
| Whiskey | 3.48 | 1.68 | 2.71 | 4.24 | 1.00 | 6.00 | 2.95 | | 3.86 | 3.62 | | 21 |  |  |
| Yogurt | 3.48 | 1.54 | 2.77 | 4.18 | 1.00 | 6.00 | 3.29 | | 3.52 | 3.62 | | 21 |  |  |
| Breakfast cereal | 3.46 | 1.33 | 2.85 | 4.07 | 1.00 | 6.00 | 3.43 | | 3.43 | 3.52 | | 21 |  |  |
| Room (renting) | 3.44 | 1.74 | 2.65 | 4.24 | 1.00 | 6.00 | 3.43 | | 3.48 | 3.43 | | 21 |  |  |
| Guitar | 3.41 | 1.62 | 2.69 | 4.13 | 1.00 | 6.00 | 3.18 | | 3.41 | 3.64 | | 22 |  |  |
| Makeup | 3.37 | 1.46 | 2.70 | 4.03 | 1.00 | 5.67 | 2.90 | | 3.38 | 3.81 | | 21 |  |  |
| Vegetables | 3.33 | 1.67 | 2.57 | 4.09 | 1.00 | 6.00 | 3.10 | | 3.57 | 3.33 | | 21 |  |  |
| CD | 3.30 | 1.36 | 2.68 | 3.92 | 1.00 | 5.33 | 2.90 | | 3.71 | 3.29 | | 21 |  |  |
| Speakers | 3.30 | 1.53 | 2.60 | 4.00 | 1.00 | 6.00 | 2.86 | | 3.57 | 3.48 | | 21 |  |  |
| 3D printer | 3.30 | 1.29 | 2.72 | 3.89 | 1.00 | 5.33 | 3.00 | | 3.24 | 3.67 | | 21 |  |  |
| Book | 3.29 | 1.55 | 2.60 | 3.98 | 1.00 | 6.00 | 2.95 | | 3.32 | 3.59 | | 22 |  |  |
| Shower gel | 3.29 | 1.61 | 2.55 | 4.02 | 1.00 | 6.00 | 3.10 | | 3.00 | 3.76 | | 21 |  |  |
| Mouthwash | 3.27 | 1.63 | 2.53 | 4.01 | 1.00 | 6.00 | 3.00 | | 3.14 | 3.67 | | 21 |  |  |
| Cigarettes | 3.27 | 1.58 | 2.55 | 3.99 | 1.00 | 6.00 | 3.05 | | 3.29 | 3.48 | | 21 |  |  |
| Shampoo | 3.27 | 1.57 | 2.56 | 3.98 | 1.00 | 6.00 | 2.86 | | 3.33 | 3.62 | | 21 |  |  |
| Piano | 3.26 | 1.50 | 2.59 | 3.92 | 1.00 | 6.00 | 2.95 | | 3.18 | 3.64 | | 22 |  |  |
| Tea | 3.24 | 1.64 | 2.51 | 3.97 | 1.00 | 6.00 | 3.00 | | 3.18 | 3.55 | | 22 |  |  |
| DVD player | 3.24 | 1.25 | 2.67 | 3.81 | 1.00 | 5.00 | 2.95 | | 3.38 | 3.38 | | 21 |  |  |
| Deodorant | 3.22 | 1.76 | 2.42 | 4.02 | 1.00 | 6.00 | 3.14 | | 3.10 | 3.43 | | 21 |  |  |
| Radio | 3.21 | 1.50 | 2.53 | 3.89 | 1.00 | 5.67 | 2.76 | | 3.33 | 3.52 | | 21 |  |  |
| Painting | 3.16 | 1.70 | 2.39 | 3.93 | 1.00 | 6.00 | 3.19 | | 3.14 | 3.14 | | 21 |  |  |
| Pillow | 3.14 | 1.48 | 2.47 | 3.82 | 1.00 | 5.33 | 3.05 | | 3.19 | 3.19 | | 21 |  |  |
| Scissors | 3.11 | 1.48 | 2.44 | 3.79 | 1.00 | 5.00 | 2.76 | | 3.05 | 3.52 | | 21 |  |  |
| Laundry detergent | 3.10 | 1.36 | 2.47 | 3.72 | 1.00 | 5.33 | 2.90 | | 3.00 | 3.38 | | 21 |  |  |
| Smartphone | 3.05 | 1.38 | 2.42 | 3.67 | 1.00 | 5.67 | 2.81 | | 3.05 | 3.29 | | 21 |  |  |
| Sunscreen | 3.03 | 1.67 | 2.27 | 3.79 | 1.00 | 6.00 | 2.76 | | 3.24 | 3.10 | | 21 |  |  |
| Milk | 2.98 | 1.60 | 2.28 | 3.69 | 1.00 | 6.00 | 2.86 | | 3.00 | 3.09 | | 22 |  |  |
| House | 2.98 | 1.42 | 2.34 | 3.63 | 1.00 | 5.00 | 3.00 | | 2.76 | 3.19 | | 21 |  |  |
| Toilet brush | 2.98 | 1.64 | 2.24 | 3.73 | 1.00 | 5.33 | 2.67 | | 2.95 | 3.33 | | 21 |  |  |
| Desktop computer | 2.97 | 1.50 | 2.29 | 3.65 | 1.00 | 6.00 | 2.43 | | 3.10 | 3.38 | | 21 |  |  |
| Perfume | 2.95 | 1.35 | 2.34 | 3.57 | 1.00 | 4.67 | 2.76 | | 3.10 | 3.00 | | 21 |  |  |
| Air conditioner | 2.95 | 1.53 | 2.26 | 3.65 | 1.00 | 5.00 | 2.76 | | 3.10 | 3.00 | | 21 |  |  |
| Television | 2.94 | 1.43 | 2.29 | 3.59 | 1.00 | 5.33 | 2.43 | | 3.24 | 3.14 | | 21 |  |  |
| Cleaning cloth | 2.94 | 1.70 | 2.16 | 3.71 | 1.00 | 6.00 | 2.86 | | 2.76 | 3.19 | | 21 |  |  |
| Couch | 2.92 | 1.39 | 2.29 | 3.56 | 1.00 | 5.00 | 2.90 | | 3.10 | 2.76 | | 21 |  |  |
| Aspirin | 2.89 | 1.64 | 2.14 | 3.63 | 1.00 | 6.00 | 2.48 | | 3.10 | 3.10 | | 21 |  |  |
| Dental floss | 2.89 | 1.33 | 2.28 | 3.49 | 1.00 | 6.00 | 2.48 | | 2.81 | 3.38 | | 21 |  |  |
| Insecticide | 2.86 | 1.70 | 2.11 | 3.62 | 1.00 | 5.67 | 2.59 | | 2.86 | 3.14 | | 22 |  |  |
| Disposable plastic cups | 2.86 | 1.31 | 2.26 | 3.46 | 1.00 | 5.00 | 2.48 | | 2.90 | 3.19 | | 21 |  |  |
| Dishwashing brush | 2.86 | 1.61 | 2.12 | 3.59 | 1.00 | 6.00 | 2.52 | | 2.81 | 3.24 | | 21 |  |  |
| Air freshener | 2.84 | 1.61 | 2.11 | 3.57 | 1.00 | 6.00 | 2.67 | | 2.95 | 2.90 | | 21 |  |  |
| Bread | 2.84 | 1.42 | 2.19 | 3.49 | 1.00 | 6.00 | 2.48 | | 3.14 | 2.90 | | 21 |  |  |
| Computer mouse | 2.81 | 1.58 | 2.09 | 3.53 | 1.00 | 6.00 | 2.38 | | 2.86 | 3.19 | | 21 |  |  |
| Calculator | 2.78 | 1.35 | 2.16 | 3.39 | 1.00 | 5.00 | 2.62 | | 2.67 | 3.05 | | 21 |  |  |
| Mattress | 2.76 | 1.51 | 2.08 | 3.45 | 1.00 | 6.00 | 2.33 | | 3.10 | 2.86 | | 21 |  |  |
| Cutlery | 2.75 | 1.45 | 2.09 | 3.41 | 1.00 | 5.00 | 2.38 | | 2.71 | 3.14 | | 21 |  |  |
| Eggs | 2.74 | 1.70 | 1.99 | 3.50 | 1.00 | 6.00 | 2.68 | | 2.82 | 2.73 | | 22 |  |  |
| Boots | 2.73 | 1.42 | 2.08 | 3.38 | 1.00 | 5.33 | 2.48 | | 2.62 | 3.10 | | 21 |  |  |
| GPS | 2.71 | 1.38 | 2.08 | 3.34 | 1.00 | 5.00 | 2.52 | | 2.71 | 2.90 | | 21 |  |  |
| Power bank | 2.71 | 1.17 | 2.18 | 3.24 | 1.00 | 4.33 | 2.62 | | 2.62 | 2.90 | | 21 |  |  |
| Motorcycle | 2.68 | 1.23 | 2.12 | 3.24 | 1.00 | 5.00 | 2.52 | | 2.76 | 2.76 | | 21 |  |  |
| Skin moisturizer | 2.68 | 1.69 | 1.93 | 3.43 | 1.00 | 6.00 | 2.27 | | 2.73 | 3.05 | | 22 |  |  |
| Desk | 2.63 | 1.26 | 2.06 | 3.21 | 1.00 | 5.00 | 2.38 | | 2.52 | 3.00 | | 21 |  |  |
| Bracelet | 2.60 | 1.69 | 1.84 | 3.37 | 1.00 | 5.00 | 2.57 | | 2.62 | 2.62 | | 21 |  |  |
| Water bottle | 2.60 | 1.52 | 1.91 | 3.29 | 1.00 | 4.67 | 2.29 | | 2.62 | 2.90 | | 21 |  |  |
| Camera | 2.59 | 1.52 | 1.92 | 3.26 | 1.00 | 6.00 | 2.45 | | 2.50 | 2.82 | | 22 |  |  |
| Blinds | 2.56 | 1.58 | 1.84 | 3.27 | 1.00 | 5.00 | 2.38 | | 2.43 | 2.86 | | 21 |  |  |
| Headphones | 2.56 | 1.30 | 1.96 | 3.15 | 1.00 | 5.67 | 2.29 | | 2.62 | 2.76 | | 21 |  |  |
| Razor blades | 2.56 | 1.34 | 1.94 | 3.17 | 1.00 | 5.00 | 2.24 | | 2.62 | 2.81 | | 21 |  |  |
| Eyeglasses | 2.54 | 1.77 | 1.73 | 3.35 | 1.00 | 6.00 | 2.48 | | 2.62 | 2.52 | | 21 |  |  |
| Life insurance | 2.51 | 1.64 | 1.76 | 3.25 | 1.00 | 6.00 | 2.62 | | 2.52 | 2.38 | | 21 |  |  |
| Soap | 2.48 | 1.54 | 1.78 | 3.18 | 1.00 | 6.00 | 2.24 | | 2.52 | 2.67 | | 21 |  |  |
| After shave | 2.47 | 1.50 | 1.80 | 3.13 | 1.00 | 5.00 | 2.23 | | 2.41 | 2.77 | | 22 |  |  |
| Laptop | 2.47 | 1.53 | 1.79 | 3.15 | 1.00 | 5.00 | 2.14 | | 2.50 | 2.77 | | 22 |  |  |
| Curtains | 2.44 | 1.41 | 1.80 | 3.09 | 1.00 | 4.67 | 2.29 | | 2.43 | 2.62 | | 21 |  |  |
| Cooking oil | 2.44 | 1.49 | 1.78 | 3.10 | 1.00 | 6.00 | 2.00 | | 2.32 | 3.00 | | 22 |  |  |
| Pants | 2.43 | 1.35 | 1.82 | 3.04 | 1.00 | 5.00 | 2.33 | | 2.29 | 2.67 | | 21 |  |  |
| Toilet paper | 2.42 | 1.27 | 1.86 | 2.99 | 1.00 | 5.00 | 1.68 | | 2.50 | 3.09 | | 22 |  |  |
| Picture frame | 2.41 | 1.49 | 1.73 | 3.09 | 1.00 | 5.33 | 2.19 | | 2.48 | 2.57 | | 21 |  |  |
| Car | 2.41 | 1.34 | 1.82 | 3.00 | 1.00 | 4.33 | 2.23 | | 2.36 | 2.64 | | 22 |  |  |
| Oven mitts | 2.40 | 1.54 | 1.70 | 3.10 | 1.00 | 5.67 | 2.05 | | 2.57 | 2.57 | | 21 |  |  |
| Bedding | 2.38 | 1.20 | 1.83 | 2.93 | 1.00 | 4.33 | 2.05 | | 2.57 | 2.52 | | 21 |  |  |
| Toothpaste | 2.38 | 1.33 | 1.79 | 2.97 | 1.00 | 5.00 | 1.95 | | 2.36 | 2.82 | | 22 |  |  |
| Postcard | 2.37 | 1.25 | 1.80 | 2.93 | 1.00 | 5.00 | 2.19 | | 2.38 | 2.52 | | 21 |  |  |
| Dish detergent | 2.36 | 1.19 | 1.83 | 2.89 | 1.00 | 5.00 | 1.73 | | 2.36 | 3.00 | | 22 |  |  |
| Tablet | 2.35 | 1.21 | 1.80 | 2.90 | 1.00 | 4.00 | 2.05 | | 2.33 | 2.67 | | 21 |  |  |
| Blanket | 2.33 | 1.25 | 1.76 | 2.90 | 1.00 | 5.00 | 2.14 | | 2.57 | 2.29 | | 21 |  |  |
| Gas outdoor grill | 2.32 | 1.39 | 1.70 | 2.94 | 1.00 | 5.33 | 2.05 | | 2.32 | 2.59 | | 22 |  |  |
| Plants | 2.30 | 1.22 | 1.75 | 2.85 | 1.00 | 4.33 | 2.00 | | 2.62 | 2.29 | | 21 |  |  |
| Hangers | 2.25 | 1.45 | 1.59 | 2.91 | 1.00 | 5.00 | 2.05 | | 2.29 | 2.43 | | 21 |  |  |
| Backpack | 2.24 | 1.14 | 1.72 | 2.76 | 1.00 | 4.33 | 1.86 | | 2.19 | 2.67 | | 21 |  |  |
| Hybrid/Convertible laptop | 2.23 | 1.49 | 1.57 | 2.89 | 1.00 | 5.00 | 2.05 | | 2.27 | 2.36 | | 22 |  |  |
| Apartment | 2.20 | 1.25 | 1.64 | 2.75 | 1.00 | 4.00 | 2.27 | | 2.18 | 2.14 | | 22 |  |  |
| Adhesive bandage | 2.14 | 1.29 | 1.57 | 2.71 | 1.00 | 5.67 | 1.86 | | 2.09 | 2.45 | | 22 |  |  |
| Glasses (drinking) | 2.13 | 1.12 | 1.62 | 2.64 | 1.00 | 4.00 | 1.90 | | 2.29 | 2.19 | | 21 |  |  |
| Toaster | 2.13 | 1.04 | 1.65 | 2.60 | 1.00 | 4.00 | 2.05 | | 2.10 | 2.24 | | 21 |  |  |
| **Suit** | 2.07 | 1.20 | 1.77 | 2.37 | 1.00 | 5.00 | 2.02 | | 2.08 | 2.13 | | 64 |  |  |
| Printer | 2.06 | 1.24 | 1.51 | 2.61 | 1.00 | 4.67 | 1.86 | | 1.95 | 2.36 | | 22 |  |  |
| Microwave | 2.06 | 1.22 | 1.52 | 2.60 | 1.00 | 4.67 | 1.55 | | 1.95 | 2.68 | | 22 |  |  |
| Baby diapers | 2.06 | 1.34 | 1.47 | 2.65 | 1.00 | 5.33 | 1.77 | | 2.09 | 2.32 | | 22 |  |  |
| Trash bag | 2.02 | 1.35 | 1.42 | 2.61 | 1.00 | 5.00 | 1.77 | | 1.95 | 2.32 | | 22 |  |  |
| Paper clips | 2.00 | 1.35 | 1.39 | 2.61 | 1.00 | 6.00 | 1.76 | | 2.00 | 2.24 | | 21 |  |  |
| Shoes | 2.00 | 1.26 | 1.44 | 2.56 | 1.00 | 4.67 | 1.86 | | 1.91 | 2.23 | | 22 |  |  |
| Vacuum cleaner | 1.98 | 1.14 | 1.48 | 2.49 | 1.00 | 5.00 | 1.45 | | 1.95 | 2.55 | | 22 |  |  |
| Sun glasses | 1.97 | 1.15 | 1.46 | 2.48 | 1.00 | 4.67 | 1.82 | | 1.95 | 2.14 | | 22 |  |  |
| Chair | 1.97 | 1.09 | 1.47 | 2.46 | 1.00 | 4.00 | 1.62 | | 2.14 | 2.14 | | 21 |  |  |
| Towel | 1.94 | 1.24 | 1.39 | 2.49 | 1.00 | 5.00 | 1.59 | | 2.05 | 2.18 | | 22 |  |  |
| Umbrella | 1.89 | 1.24 | 1.32 | 2.45 | 1.00 | 5.00 | 1.76 | | 1.81 | 2.10 | | 21 |  |  |
| Notebook | 1.88 | 0.92 | 1.47 | 2.29 | 1.00 | 3.67 | 1.59 | | 1.82 | 2.23 | | 22 |  |  |
| Dress | 1.86 | 1.11 | 1.37 | 2.35 | 1.00 | 3.67 | 1.64 | | 1.91 | 2.05 | | 22 |  |  |
| USB flash drive | 1.85 | 1.18 | 1.33 | 2.37 | 1.00 | 5.00 | 1.59 | | 1.77 | 2.18 | | 22 |  |  |
| Pajamas | 1.85 | 1.02 | 1.40 | 2.30 | 1.00 | 4.33 | 1.64 | | 1.82 | 2.09 | | 22 |  |  |
| **Necklace** | 1.84 | 1.15 | 1.55 | 2.13 | 1.00 | 5.00 | 1.86 | | 1.88 | 1.78 | | 64 |  |  |
| Pen | 1.83 | 1.06 | 1.36 | 2.30 | 1.00 | 4.00 | 1.68 | | 1.73 | 2.09 | | 22 |  |  |
| Coasters | 1.83 | 1.03 | 1.36 | 2.29 | 1.00 | 4.00 | 1.81 | | 1.76 | 1.90 | | 21 |  |  |
| **Vase** | 1.79 | 1.10 | 1.52 | 2.07 | 1.00 | 4.67 | 1.78 | | 1.80 | 1.80 | | 64 |  |  |
| T-shirt | 1.76 | 0.95 | 1.33 | 2.19 | 1.00 | 4.00 | 1.57 | | 1.86 | 1.86 | | 21 |  |  |
| Ceramic mug | 1.76 | 1.09 | 1.27 | 2.24 | 1.00 | 4.67 | 1.50 | | 1.64 | 2.14 | | 22 |  |  |
| Watch | 1.73 | 0.95 | 1.31 | 2.15 | 1.00 | 3.67 | 1.50 | | 1.73 | 1.95 | | 22 |  |  |
| Flashlight | 1.73 | 1.33 | 1.14 | 2.32 | 1.00 | 6.00 | 1.55 | | 1.77 | 1.86 | | 22 |  |  |
| Shirt | 1.71 | 1.04 | 1.25 | 2.17 | 1.00 | 4.33 | 1.55 | | 1.73 | 1.86 | | 22 |  |  |
| Laptop bag | 1.70 | 1.11 | 1.20 | 2.19 | 1.00 | 4.67 | 1.55 | | 1.68 | 1.86 | | 22 |  |  |
| Table | 1.70 | 1.13 | 1.20 | 2.20 | 1.00 | 4.33 | 1.68 | | 1.68 | 1.73 | | 22 |  |  |
| Underwear | 1.59 | 1.00 | 1.15 | 2.04 | 1.00 | 4.67 | 1.36 | | 1.59 | 1.82 | | 22 |  |  |
| Wall clock | 1.53 | 0.88 | 1.14 | 1.92 | 1.00 | 3.67 | 1.36 | | 1.45 | 1.77 | | 22 |  |  |
| Trash bin | 1.52 | 0.77 | 1.17 | 1.86 | 1.00 | 3.67 | 1.18 | | 1.41 | 1.95 | | 22 |  |  |

Table 4. Descriptive statistics for product’s familiarity (ordered in descending order).

|  |  |  |  |  |  |  |  | **Items** | | |  |  | |  |
| --- | --- | --- | --- | --- | --- | --- | --- | --- | --- | --- | --- | --- | --- | --- |
| **Product** | **Mean (across 3 items)** | **SD** | **CI -95** | **CI 95** | **Min** | **Max** | **1. How familiar are you with this product?** | | **2. How familiar are you with the features of this product?** | **3. How frequently do you buy this product?** | | | **n** | |
| Toilet paper | 5.62 | 0.69 | 5.32 | 5.92 | 3.33 | 6.00 | 5.57 | | 5.74 | 5.57 | | | 23 | |
| Bread | 5.61 | 0.60 | 5.35 | 5.87 | 4.00 | 6.00 | 5.74 | | 5.70 | 5.39 | | | 23 | |
| Pizza | 5.58 | 0.60 | 5.32 | 5.84 | 4.00 | 6.00 | 5.65 | | 5.83 | 5.26 | | | 23 | |
| Soap | 5.54 | 0.72 | 5.23 | 5.85 | 3.67 | 6.00 | 5.65 | | 5.70 | 5.26 | | | 23 | |
| Toothpaste | 5.43 | 0.80 | 5.09 | 5.77 | 2.33 | 6.00 | 5.67 | | 5.79 | 4.83 | | | 24 | |
| Deodorant | 5.43 | 0.98 | 5.02 | 5.84 | 1.67 | 6.00 | 5.58 | | 5.67 | 5.04 | | | 24 | |
| Pen | 5.36 | 0.67 | 5.08 | 5.64 | 4.00 | 6.00 | 5.71 | | 5.67 | 4.71 | | | 24 | |
| **Shampoo** | 5.36 | 0.89 | 5.15 | 5.57 | 1.00 | 6.00 | 5.54 | | 5.49 | 5.04 | | | 71 | |
| Cookies | 5.35 | 0.84 | 4.99 | 5.71 | 3.00 | 6.00 | 5.65 | | 5.61 | 4.78 | | | 23 | |
| Eggs | 5.32 | 0.79 | 4.99 | 5.65 | 3.33 | 6.00 | 5.63 | | 5.58 | 4.75 | | | 24 | |
| Fruit | 5.32 | 1.02 | 4.89 | 5.75 | 2.00 | 6.00 | 5.46 | | 5.38 | 5.13 | | | 24 | |
| Vegetables | 5.31 | 1.06 | 4.86 | 5.75 | 2.33 | 6.00 | 5.46 | | 5.29 | 5.17 | | | 24 | |
| Laundry detergent | 5.30 | 0.83 | 4.94 | 5.67 | 3.67 | 6.00 | 5.52 | | 5.61 | 4.78 | | | 23 | |
| Ice cream | 5.29 | 0.83 | 4.93 | 5.65 | 3.00 | 6.00 | 5.52 | | 5.61 | 4.74 | | | 23 | |
| Book | 5.28 | 0.68 | 4.99 | 5.56 | 3.67 | 6.00 | 5.92 | | 5.63 | 4.29 | | | 24 | |
| Trash bag | 5.28 | 0.75 | 4.96 | 5.60 | 4.00 | 6.00 | 5.54 | | 5.50 | 4.79 | | | 24 | |
| T-shirt | 5.28 | 0.49 | 5.06 | 5.49 | 4.00 | 6.00 | 5.65 | | 5.83 | 4.35 | | | 23 | |
| Cooking oil | 5.25 | 0.84 | 4.88 | 5.61 | 3.00 | 6.00 | 5.61 | | 5.52 | 4.61 | | | 23 | |
| Yogurt | 5.25 | 1.23 | 4.72 | 5.78 | 1.00 | 6.00 | 5.35 | | 5.39 | 5.00 | | | 23 | |
| Water bottle | 5.24 | 0.87 | 4.87 | 5.60 | 3.00 | 6.00 | 5.67 | | 5.75 | 4.29 | | | 24 | |
| Underwear | 5.23 | 0.66 | 4.95 | 5.52 | 3.33 | 6.00 | 5.70 | | 5.65 | 4.35 | | | 23 | |
| **Breakfast cereal** | 5.22 | 0.96 | 4.99 | 5.44 | 1.00 | 6.00 | 5.54 | | 5.38 | 4.73 | | | 71 | |
| Pants | 5.17 | 0.82 | 4.82 | 5.51 | 3.00 | 6.00 | 5.58 | | 5.58 | 4.33 | | | 24 | |
| Milk | 5.15 | 1.01 | 4.73 | 5.58 | 2.00 | 6.00 | 5.46 | | 5.67 | 4.33 | | | 24 | |
| Dish detergent | 5.14 | 0.79 | 4.80 | 5.47 | 3.00 | 6.00 | 5.46 | | 5.50 | 4.46 | | | 24 | |
| Peanut butter | 5.13 | 0.93 | 4.73 | 5.53 | 2.67 | 6.00 | 5.48 | | 5.52 | 4.39 | | | 23 | |
| Shoes | 5.10 | 0.55 | 4.87 | 5.34 | 4.33 | 6.00 | 5.78 | | 5.65 | 3.87 | | | 23 | |
| Shirt | 5.10 | 0.81 | 4.75 | 5.44 | 3.33 | 6.00 | 5.42 | | 5.50 | 4.38 | | | 24 | |
| Dental floss | 5.07 | 0.74 | 4.76 | 5.38 | 3.33 | 6.00 | 5.54 | | 5.42 | 4.25 | | | 24 | |
| Entrée at a restaurant | 5.07 | 0.79 | 4.74 | 5.40 | 3.33 | 6.00 | 5.71 | | 5.50 | 4.00 | | | 24 | |
| Potato chips | 5.00 | 0.93 | 4.61 | 5.39 | 3.00 | 6.00 | 5.50 | | 5.38 | 4.13 | | | 24 | |
| Soft drink | 4.96 | 1.11 | 4.49 | 5.43 | 3.00 | 6.00 | 5.21 | | 5.38 | 4.29 | | | 24 | |
| Shower gel | 4.96 | 0.98 | 4.53 | 5.38 | 3.00 | 6.00 | 5.48 | | 5.35 | 4.04 | | | 23 | |
| Air freshener | 4.92 | 1.15 | 4.43 | 5.40 | 1.33 | 6.00 | 5.46 | | 5.25 | 4.04 | | | 24 | |
| Coffee | 4.90 | 1.46 | 4.29 | 5.52 | 1.67 | 6.00 | 5.17 | | 5.08 | 4.46 | | | 24 | |
| Appetizers | 4.90 | 0.95 | 4.50 | 5.30 | 3.33 | 6.00 | 5.58 | | 5.42 | 3.71 | | | 24 | |
| Chocolate bar | 4.88 | 1.30 | 4.32 | 5.43 | 1.00 | 6.00 | 5.42 | | 5.46 | 3.75 | | | 24 | |
| Mouthwash | 4.87 | 1.11 | 4.39 | 5.35 | 2.33 | 6.00 | 5.26 | | 5.26 | 4.09 | | | 23 | |
| Pillow | 4.85 | 0.50 | 4.64 | 5.06 | 4.00 | 6.00 | 5.71 | | 5.67 | 3.17 | | | 24 | |
| Blanket | 4.85 | 0.80 | 4.51 | 5.18 | 3.00 | 6.00 | 5.67 | | 5.58 | 3.29 | | | 24 | |
| Internet provider | 4.83 | 1.15 | 4.35 | 5.32 | 2.33 | 6.00 | 5.21 | | 5.08 | 4.21 | | | 24 | |
| **Chewing gum** | 4.83 | 0.90 | 4.62 | 5.04 | 2.33 | 6.00 | 5.51 | | 5.32 | 3.66 | | | 71 | |
| Eyeglasses | 4.83 | 0.78 | 4.49 | 5.16 | 3.00 | 6.00 | 5.57 | | 5.52 | 3.39 | | | 23 | |
| Sun glasses | 4.83 | 0.74 | 4.50 | 5.15 | 3.33 | 6.00 | 5.57 | | 5.65 | 3.26 | | | 23 | |
| Dinner at a restaurant | 4.82 | 1.04 | 4.38 | 5.26 | 2.33 | 6.00 | 5.38 | | 5.29 | 3.79 | | | 24 | |
| Towel | 4.77 | 0.73 | 4.45 | 5.09 | 3.00 | 6.00 | 5.57 | | 5.43 | 3.30 | | | 23 | |
| Headphones | 4.76 | 0.96 | 4.36 | 5.17 | 1.67 | 6.00 | 5.42 | | 5.46 | 3.42 | | | 24 | |
| Tea | 4.75 | 1.26 | 4.22 | 5.28 | 1.00 | 6.00 | 5.13 | | 5.13 | 4.00 | | | 24 | |
| Movie ticket | 4.74 | 0.97 | 4.32 | 5.16 | 2.00 | 6.00 | 5.39 | | 5.48 | 3.35 | | | 23 | |
| Table | 4.74 | 0.85 | 4.38 | 5.10 | 3.00 | 6.00 | 5.67 | | 5.46 | 3.08 | | | 24 | |
| Razor blades | 4.72 | 1.15 | 4.24 | 5.21 | 1.33 | 6.00 | 5.29 | | 5.08 | 3.79 | | | 24 | |
| Hotel room | 4.71 | 0.89 | 4.33 | 5.08 | 2.33 | 6.00 | 5.42 | | 5.50 | 3.21 | | | 24 | |
| Trash bin | 4.71 | 0.82 | 4.36 | 5.06 | 1.67 | 6.00 | 5.42 | | 5.42 | 3.29 | | | 24 | |
| Cleaning cloth | 4.69 | 0.99 | 4.28 | 5.11 | 2.67 | 6.00 | 5.33 | | 5.17 | 3.58 | | | 24 | |
| Online streaming subscription | 4.68 | 1.21 | 4.17 | 5.19 | 1.67 | 6.00 | 5.25 | | 4.96 | 3.83 | | | 24 | |
| Sunscreen | 4.68 | 1.19 | 4.18 | 5.18 | 1.00 | 6.00 | 5.33 | | 5.21 | 3.50 | | | 24 | |
| Car | 4.67 | 0.90 | 4.29 | 5.05 | 2.33 | 6.00 | 5.54 | | 5.38 | 3.08 | | | 24 | |
| DVD | 4.67 | 0.72 | 4.36 | 4.98 | 2.67 | 6.00 | 5.43 | | 5.61 | 2.96 | | | 23 | |
| Glasses (drinking) | 4.67 | 0.88 | 4.29 | 5.04 | 2.67 | 6.00 | 5.58 | | 5.21 | 3.21 | | | 24 | |
| Chair | 4.65 | 1.03 | 4.22 | 5.09 | 2.33 | 6.00 | 5.54 | | 5.29 | 3.13 | | | 24 | |
| Wall clock | 4.65 | 0.62 | 4.39 | 4.91 | 3.67 | 6.00 | 5.83 | | 5.42 | 2.71 | | | 24 | |
| Computer mouse | 4.65 | 0.68 | 4.37 | 4.94 | 3.67 | 6.00 | 5.63 | | 5.38 | 2.96 | | | 24 | |
| Adhesive bandage | 4.65 | 1.03 | 4.21 | 5.10 | 1.67 | 6.00 | 5.30 | | 5.26 | 3.39 | | | 23 | |
| Boots | 4.64 | 0.98 | 4.22 | 5.05 | 1.33 | 6.00 | 5.46 | | 5.33 | 3.13 | | | 24 | |
| Television | 4.63 | 0.89 | 4.25 | 5.00 | 1.67 | 6.00 | 5.63 | | 5.38 | 2.88 | | | 24 | |
| Printer | 4.63 | 0.65 | 4.35 | 4.90 | 3.67 | 6.00 | 5.67 | | 5.54 | 2.67 | | | 24 | |
| Skin moisturizer | 4.58 | 1.36 | 4.01 | 5.16 | 1.00 | 6.00 | 5.21 | | 5.00 | 3.54 | | | 24 | |
| Notebook | 4.57 | 0.97 | 4.16 | 4.98 | 2.33 | 6.00 | 5.25 | | 5.04 | 3.42 | | | 24 | |
| Smartphone | 4.57 | 1.11 | 4.10 | 5.04 | 1.67 | 6.00 | 5.13 | | 5.25 | 3.33 | | | 24 | |
| Toilet brush | 4.56 | 0.95 | 4.15 | 4.96 | 2.00 | 6.00 | 5.46 | | 5.33 | 2.88 | | | 24 | |
| Toaster | 4.54 | 0.82 | 4.19 | 4.89 | 2.33 | 6.00 | 5.46 | | 5.21 | 2.96 | | | 24 | |
| Radio | 4.54 | 0.90 | 4.16 | 4.92 | 1.67 | 6.00 | 5.54 | | 5.58 | 2.50 | | | 24 | |
| Dessert at a restaurant | 4.53 | 1.19 | 4.03 | 5.03 | 1.67 | 6.00 | 5.29 | | 5.13 | 3.17 | | | 24 | |
| Pajamas | 4.51 | 0.93 | 4.10 | 4.91 | 2.33 | 6.00 | 5.30 | | 5.26 | 2.96 | | | 23 | |
| Scissors | 4.50 | 0.86 | 4.14 | 4.86 | 1.67 | 5.67 | 5.25 | | 5.21 | 3.04 | | | 24 | |
| CD | 4.50 | 1.03 | 4.07 | 4.93 | 2.33 | 6.00 | 5.29 | | 5.17 | 3.04 | | | 24 | |
| Paper clips | 4.49 | 0.87 | 4.12 | 4.85 | 2.33 | 6.00 | 5.38 | | 5.21 | 2.88 | | | 24 | |
| Mattress | 4.49 | 0.47 | 4.29 | 4.69 | 3.67 | 6.00 | 5.58 | | 5.33 | 2.54 | | | 24 | |
| Aspirin | 4.49 | 1.07 | 4.04 | 4.94 | 1.67 | 6.00 | 5.33 | | 5.17 | 2.96 | | | 24 | |
| Flashlight | 4.48 | 0.78 | 4.14 | 4.81 | 2.67 | 6.00 | 5.30 | | 5.30 | 2.83 | | | 23 | |
| Hangers | 4.45 | 0.73 | 4.13 | 4.76 | 3.00 | 6.00 | 5.52 | | 5.30 | 2.52 | | | 23 | |
| Disposable plastic cups | 4.44 | 1.06 | 4.00 | 4.89 | 2.33 | 6.00 | 5.33 | | 5.17 | 2.83 | | | 24 | |
| Beer | 4.43 | 1.23 | 3.90 | 4.97 | 1.67 | 6.00 | 5.04 | | 5.04 | 3.22 | | | 23 | |
| Oven mitts | 4.43 | 0.83 | 4.08 | 4.78 | 2.00 | 6.00 | 5.33 | | 5.13 | 2.83 | | | 24 | |
| Couch | 4.42 | 0.62 | 4.15 | 4.69 | 3.33 | 5.33 | 5.35 | | 5.35 | 2.57 | | | 23 | |
| Bedding | 4.42 | 1.05 | 3.97 | 4.86 | 1.00 | 6.00 | 5.17 | | 5.00 | 3.08 | | | 24 | |
| Curtains | 4.40 | 0.86 | 4.04 | 4.77 | 2.67 | 6.00 | 5.33 | | 5.00 | 2.88 | | | 24 | |
| Vacuum cleaner | 4.38 | 0.95 | 3.97 | 4.78 | 2.67 | 6.00 | 5.25 | | 4.79 | 3.08 | | | 24 | |
| Laptop | 4.35 | 0.82 | 3.99 | 4.70 | 1.67 | 5.33 | 5.43 | | 5.04 | 2.57 | | | 23 | |
| Microwave | 4.32 | 1.01 | 3.89 | 4.75 | 2.33 | 6.00 | 5.25 | | 4.92 | 2.79 | | | 24 | |
| Backpack | 4.30 | 1.23 | 3.77 | 4.83 | 1.00 | 6.00 | 5.04 | | 5.17 | 2.70 | | | 23 | |
| USB flash drive | 4.29 | 1.14 | 3.81 | 4.77 | 2.00 | 6.00 | 4.92 | | 4.79 | 3.17 | | | 24 | |
| Umbrella | 4.29 | 0.86 | 3.92 | 4.66 | 1.33 | 5.33 | 5.48 | | 5.26 | 2.13 | | | 23 | |
| Watch | 4.26 | 1.09 | 3.80 | 4.72 | 2.33 | 6.00 | 5.00 | | 5.00 | 2.79 | | | 24 | |
| DVD player | 4.26 | 0.96 | 3.86 | 4.67 | 1.00 | 6.00 | 5.21 | | 5.04 | 2.54 | | | 24 | |
| Concert ticket | 4.25 | 1.00 | 3.83 | 4.67 | 1.67 | 6.00 | 5.04 | | 4.92 | 2.79 | | | 24 | |
| House | 4.23 | 0.80 | 3.89 | 4.58 | 2.33 | 5.67 | 5.30 | | 5.22 | 2.17 | | | 23 | |
| Speakers | 4.22 | 1.03 | 3.79 | 4.66 | 1.67 | 6.00 | 4.92 | | 5.04 | 2.71 | | | 24 | |
| Desk | 4.22 | 0.74 | 3.90 | 4.54 | 2.00 | 5.33 | 5.30 | | 5.17 | 2.17 | | | 23 | |
| Tablet | 4.22 | 0.83 | 3.86 | 4.58 | 2.33 | 5.33 | 5.00 | | 5.09 | 2.57 | | | 23 | |
| Air conditioner | 4.21 | 0.87 | 3.84 | 4.57 | 2.33 | 6.00 | 5.08 | | 5.04 | 2.50 | | | 24 | |
| Plane ticket | 4.19 | 1.08 | 3.74 | 4.65 | 2.00 | 6.00 | 4.92 | | 4.75 | 2.92 | | | 24 | |
| Plants | 4.19 | 1.17 | 3.68 | 4.69 | 2.00 | 6.00 | 5.04 | | 4.74 | 2.78 | | | 23 | |
| Ceramic mug | 4.18 | 1.01 | 3.76 | 4.61 | 2.00 | 6.00 | 5.00 | | 4.71 | 2.83 | | | 24 | |
| Postcard | 4.17 | 1.06 | 3.72 | 4.62 | 1.67 | 6.00 | 5.17 | | 4.96 | 2.38 | | | 24 | |
| GPS | 4.15 | 1.12 | 3.68 | 4.63 | 2.33 | 6.00 | 5.08 | | 4.71 | 2.67 | | | 24 | |
| Dishwashing brush | 4.13 | 1.26 | 3.59 | 4.66 | 1.67 | 6.00 | 4.63 | | 4.71 | 3.04 | | | 24 | |
| Bicycle | 4.11 | 0.74 | 3.80 | 4.42 | 1.67 | 5.00 | 5.33 | | 5.00 | 2.00 | | | 24 | |
| Room (renting) | 4.11 | 1.45 | 3.50 | 4.72 | 1.00 | 6.00 | 4.83 | | 4.75 | 2.75 | | | 24 | |
| Suit | 4.11 | 1.31 | 3.56 | 4.66 | 1.00 | 6.00 | 4.96 | | 4.79 | 2.58 | | | 24 | |
| Necklace | 4.09 | 1.02 | 3.65 | 4.53 | 1.00 | 6.00 | 5.00 | | 4.96 | 2.30 | | | 23 | |
| Coasters | 4.08 | 1.14 | 3.60 | 4.56 | 1.00 | 6.00 | 5.00 | | 4.75 | 2.50 | | | 24 | |
| Medical treatment | 4.07 | 0.96 | 3.66 | 4.47 | 2.67 | 6.00 | 4.83 | | 4.46 | 2.92 | | | 24 | |
| Cutlery | 4.07 | 1.18 | 3.57 | 4.57 | 1.00 | 6.00 | 4.79 | | 4.63 | 2.79 | | | 24 | |
| Calculator | 4.06 | 0.94 | 3.65 | 4.46 | 1.33 | 6.00 | 5.09 | | 5.00 | 2.09 | | | 23 | |
| Desktop computer | 4.06 | 0.81 | 3.71 | 4.41 | 2.33 | 5.00 | 5.04 | | 5.09 | 2.04 | | | 23 | |
| After shave | 4.06 | 1.68 | 3.35 | 4.76 | 1.00 | 6.00 | 4.67 | | 4.54 | 2.96 | | | 24 | |
| Picture frame | 3.96 | 1.52 | 3.31 | 4.60 | 1.00 | 6.00 | 4.67 | | 4.63 | 2.58 | | | 24 | |
| Dress | 3.96 | 1.49 | 3.31 | 4.60 | 1.00 | 5.67 | 4.70 | | 4.70 | 2.48 | | | 23 | |
| Bracelet | 3.94 | 1.20 | 3.42 | 4.46 | 1.00 | 6.00 | 4.83 | | 4.87 | 2.13 | | | 23 | |
| Laptop bag | 3.93 | 1.02 | 3.50 | 4.36 | 1.00 | 6.00 | 4.79 | | 4.88 | 2.13 | | | 24 | |
| Wine | 3.92 | 1.62 | 3.23 | 4.60 | 1.00 | 6.00 | 4.50 | | 4.29 | 2.96 | | | 24 | |
| Camera | 3.90 | 0.88 | 3.52 | 4.28 | 1.33 | 5.33 | 4.96 | | 4.74 | 2.00 | | | 23 | |
| Perfume | 3.89 | 1.20 | 3.38 | 4.40 | 1.67 | 6.00 | 4.83 | | 4.33 | 2.50 | | | 24 | |
| Vase | 3.88 | 1.17 | 3.38 | 4.37 | 1.00 | 6.00 | 4.88 | | 4.54 | 2.21 | | | 24 | |
| Blinds | 3.87 | 1.27 | 3.32 | 4.42 | 1.00 | 5.33 | 5.00 | | 4.65 | 1.96 | | | 23 | |
| Makeup | 3.85 | 1.40 | 3.26 | 4.44 | 1.67 | 6.00 | 4.75 | | 4.08 | 2.71 | | | 24 | |
| Gym subscription | 3.84 | 1.14 | 3.35 | 4.33 | 1.33 | 5.33 | 4.70 | | 4.70 | 2.13 | | | 23 | |
| Whiskey | 3.81 | 1.57 | 3.14 | 4.47 | 1.00 | 6.00 | 4.46 | | 4.29 | 2.67 | | | 24 | |
| Baby diapers | 3.76 | 1.23 | 3.24 | 4.28 | 1.00 | 6.00 | 4.83 | | 4.54 | 1.92 | | | 24 | |
| Piano | 3.75 | 1.15 | 3.27 | 4.23 | 1.00 | 6.00 | 4.79 | | 4.54 | 1.92 | | | 24 | |
| Museum ticket | 3.71 | 1.39 | 3.12 | 4.30 | 1.00 | 6.00 | 4.42 | | 4.17 | 2.54 | | | 24 | |
| Apartment | 3.70 | 1.09 | 3.23 | 4.17 | 1.00 | 5.33 | 4.74 | | 4.65 | 1.70 | | | 23 | |
| PC game | 3.68 | 1.59 | 2.99 | 4.37 | 1.00 | 6.00 | 4.52 | | 4.22 | 2.30 | | | 23 | |
| Painting | 3.67 | 1.17 | 3.16 | 4.17 | 1.00 | 5.00 | 4.48 | | 4.35 | 2.17 | | | 23 | |
| **Life insurance** | 3.67 | 1.28 | 3.36 | 3.97 | 1.00 | 6.00 | 4.34 | | 3.96 | 2.70 | | | 71 | |
| Cigarettes | 3.64 | 1.72 | 2.91 | 4.36 | 1.00 | 6.00 | 4.33 | | 4.33 | 2.25 | | | 24 | |
| Gas outdoor grill | 3.63 | 1.26 | 3.09 | 4.16 | 1.00 | 6.00 | 4.63 | | 4.04 | 2.21 | | | 24 | |
| Fairground ticket | 3.58 | 1.15 | 3.10 | 4.07 | 1.67 | 6.00 | 4.38 | | 3.96 | 2.42 | | | 24 | |
| Hybrid/Convertible laptop | 3.42 | 1.36 | 2.84 | 3.99 | 1.00 | 6.00 | 3.96 | | 4.04 | 2.25 | | | 24 | |
| Vacation package | 3.42 | 1.42 | 2.82 | 4.02 | 1.00 | 6.00 | 4.13 | | 3.96 | 2.17 | | | 24 | |
| Insecticide | 3.42 | 1.23 | 2.90 | 3.94 | 1.00 | 5.00 | 4.08 | | 3.88 | 2.29 | | | 24 | |
| Massage | 3.40 | 1.19 | 2.90 | 3.90 | 1.00 | 5.00 | 4.29 | | 4.04 | 1.88 | | | 24 | |
| Guitar | 3.32 | 1.48 | 2.68 | 3.96 | 1.00 | 5.00 | 4.48 | | 3.91 | 1.57 | | | 23 | |
| Contact lenses | 3.19 | 1.63 | 2.50 | 3.88 | 1.00 | 6.00 | 3.83 | | 3.63 | 2.13 | | | 24 | |
| Motorcycle | 3.06 | 1.32 | 2.49 | 3.63 | 1.00 | 4.67 | 4.30 | | 3.52 | 1.35 | | | 23 | |
| **Cruise trip** | 2.81 | 1.32 | 2.49 | 3.12 | 1.00 | 5.33 | 3.24 | | 3.41 | 1.77 | | | 71 | |
| Nature park ticket | 2.80 | 1.43 | 2.18 | 3.42 | 1.00 | 6.00 | 3.30 | | 3.22 | 1.87 | | | 23 | |
| **3D printer** | 2.56 | 1.23 | 2.27 | 2.85 | 1.00 | 5.67 | 3.17 | | 2.92 | 1.59 | | | 71 | |
| Power bank | 2.41 | 1.64 | 1.70 | 3.11 | 1.00 | 5.00 | 2.87 | | 2.78 | 1.57 | | | 23 | |

Table 5. Descriptive statistics for product’s perceived price (ordered in descending order).

|  |  |  |  |  |  |  |  | **Items** | | |  |  | |  |
| --- | --- | --- | --- | --- | --- | --- | --- | --- | --- | --- | --- | --- | --- | --- |
| **Product** | **Mean (across 3 items)** | **SD** | **CI -95** | **CI 95** | **Min** | **Max** | **1. How expensive is this product?** | | **2. How pricey is this product?** | **3. How high is this product’s price?** | | | **n** | |
| **House** | 5.48 | 0.94 | 5.25 | 5.71 | 2.67 | 6.00 | 5.45 | | 5.47 | 5.52 | | | 66 | |
| **Car** | 5.11 | 0.89 | 4.89 | 5.33 | 3.00 | 6.00 | 5.08 | | 5.15 | 5.11 | | | 66 | |
| Medical treatment | 4.90 | 1.03 | 4.43 | 5.38 | 3.00 | 6.00 | 4.86 | | 4.90 | 4.95 | | | 21 | |
| **Cruise trip** | 4.76 | 0.94 | 4.53 | 4.99 | 3.00 | 6.00 | 4.61 | | 4.80 | 4.88 | | | 66 | |
| Vacation package | 4.73 | 0.71 | 4.41 | 5.05 | 3.00 | 6.00 | 4.62 | | 4.76 | 4.81 | | | 21 | |
| Piano | 4.51 | 0.74 | 4.17 | 4.85 | 3.00 | 6.00 | 4.43 | | 4.57 | 4.52 | | | 21 | |
| Hybrid/Convertible laptop | 4.46 | 1.10 | 3.99 | 4.92 | 1.00 | 6.00 | 4.38 | | 4.46 | 4.54 | | | 24 | |
| Apartment | 4.35 | 0.73 | 4.04 | 4.66 | 3.00 | 6.00 | 4.21 | | 4.46 | 4.38 | | | 24 | |
| Motorcycle | 4.28 | 0.98 | 3.87 | 4.69 | 1.00 | 6.00 | 4.33 | | 4.17 | 4.33 | | | 24 | |
| 3D printer | 4.25 | 1.00 | 3.83 | 4.67 | 3.00 | 6.00 | 4.17 | | 4.25 | 4.33 | | | 24 | |
| Plane ticket | 4.24 | 0.87 | 3.87 | 4.60 | 2.67 | 6.00 | 4.25 | | 4.21 | 4.25 | | | 24 | |
| Desktop computer | 4.22 | 0.66 | 3.92 | 4.52 | 3.00 | 5.00 | 4.33 | | 4.05 | 4.29 | | | 21 | |
| Laptop | 4.16 | 0.90 | 3.75 | 4.57 | 3.00 | 6.00 | 4.10 | | 4.14 | 4.24 | | | 21 | |
| Life insurance | 4.06 | 1.25 | 3.50 | 4.63 | 2.00 | 6.00 | 4.05 | | 4.00 | 4.14 | | | 21 | |
| Suit | 4.03 | 1.11 | 3.56 | 4.50 | 2.00 | 6.00 | 3.83 | | 4.00 | 4.25 | | | 24 | |
| Hotel room | 4.00 | 0.92 | 3.61 | 4.39 | 2.00 | 6.00 | 3.92 | | 4.00 | 4.08 | | | 24 | |
| Room (renting) | 4.00 | 0.77 | 3.65 | 4.35 | 3.00 | 6.00 | 4.00 | | 3.95 | 4.05 | | | 21 | |
| Couch | 3.93 | 0.74 | 3.62 | 4.24 | 3.00 | 6.00 | 3.75 | | 3.96 | 4.08 | | | 24 | |
| Smartphone | 3.92 | 1.07 | 3.43 | 4.41 | 2.00 | 6.00 | 3.86 | | 3.90 | 4.00 | | | 21 | |
| Air conditioner | 3.83 | 0.83 | 3.45 | 4.20 | 2.67 | 6.00 | 3.71 | | 3.90 | 3.86 | | | 21 | |
| Television | 3.79 | 0.81 | 3.42 | 4.16 | 2.00 | 5.00 | 3.81 | | 3.81 | 3.76 | | | 21 | |
| Necklace | 3.74 | 1.24 | 3.21 | 4.26 | 1.00 | 6.00 | 3.46 | | 3.79 | 3.96 | | | 24 | |
| Camera | 3.72 | 1.13 | 3.24 | 4.20 | 2.00 | 6.00 | 3.67 | | 3.75 | 3.75 | | | 24 | |
| Painting | 3.64 | 1.16 | 3.15 | 4.13 | 1.67 | 6.00 | 3.38 | | 3.75 | 3.79 | | | 24 | |
| Watch | 3.63 | 0.86 | 3.25 | 4.02 | 2.00 | 6.00 | 3.57 | | 3.62 | 3.71 | | | 21 | |
| Vacuum cleaner | 3.58 | 1.06 | 3.13 | 4.03 | 2.00 | 6.00 | 3.50 | | 3.71 | 3.54 | | | 24 | |
| Tablet | 3.56 | 0.96 | 3.12 | 3.99 | 1.67 | 5.00 | 3.48 | | 3.62 | 3.57 | | | 21 | |
| Mattress | 3.49 | 0.89 | 3.09 | 3.90 | 2.00 | 6.00 | 3.43 | | 3.52 | 3.52 | | | 21 | |
| Guitar | 3.46 | 1.09 | 2.96 | 3.96 | 2.00 | 6.00 | 3.43 | | 3.48 | 3.48 | | | 21 | |
| Speakers | 3.43 | 1.23 | 2.91 | 3.95 | 1.67 | 6.00 | 3.33 | | 3.63 | 3.33 | | | 24 | |
| Desk | 3.40 | 1.03 | 2.93 | 3.86 | 2.00 | 6.00 | 3.24 | | 3.38 | 3.57 | | | 21 | |
| Perfume | 3.39 | 1.17 | 2.90 | 3.88 | 1.00 | 6.00 | 3.25 | | 3.58 | 3.33 | | | 24 | |
| Table | 3.35 | 0.91 | 2.96 | 3.73 | 1.00 | 5.33 | 3.38 | | 3.33 | 3.33 | | | 24 | |
| Bicycle | 3.35 | 1.19 | 2.84 | 3.85 | 1.00 | 6.00 | 3.29 | | 3.33 | 3.42 | | | 24 | |
| Gas outdoor grill | 3.33 | 0.61 | 3.06 | 3.61 | 2.00 | 4.00 | 3.33 | | 3.29 | 3.38 | | | 21 | |
| Internet provider | 3.30 | 1.09 | 2.80 | 3.80 | 2.00 | 6.00 | 3.24 | | 3.38 | 3.29 | | | 21 | |
| GPS | 3.22 | 1.06 | 2.74 | 3.71 | 1.00 | 5.00 | 3.24 | | 3.29 | 3.14 | | | 21 | |
| Concert ticket | 3.21 | 1.07 | 2.72 | 3.69 | 1.00 | 6.00 | 3.10 | | 3.29 | 3.24 | | | 21 | |
| Eyeglasses | 3.17 | 1.09 | 2.68 | 3.67 | 2.00 | 6.00 | 3.14 | | 3.19 | 3.19 | | | 21 | |
| Bracelet | 3.13 | 0.92 | 2.71 | 3.55 | 2.00 | 5.00 | 3.05 | | 3.10 | 3.24 | | | 21 | |
| Headphones | 3.13 | 1.08 | 2.67 | 3.58 | 1.00 | 5.00 | 2.92 | | 3.25 | 3.21 | | | 24 | |
| Printer | 3.08 | 0.82 | 2.71 | 3.45 | 2.00 | 5.00 | 3.05 | | 3.05 | 3.14 | | | 21 | |
| PC game | 3.03 | 1.18 | 2.53 | 3.53 | 1.00 | 6.00 | 2.83 | | 3.21 | 3.04 | | | 24 | |
| Boots | 3.01 | 1.22 | 2.50 | 3.53 | 1.00 | 6.00 | 2.88 | | 3.08 | 3.08 | | | 24 | |
| Sun glasses | 3.01 | 1.53 | 2.37 | 3.66 | 1.00 | 6.00 | 2.83 | | 3.04 | 3.17 | | | 24 | |
| Bedding | 2.99 | 0.96 | 2.58 | 3.39 | 1.00 | 4.67 | 2.83 | | 3.13 | 3.00 | | | 24 | |
| Radio | 2.96 | 1.16 | 2.47 | 3.45 | 1.67 | 6.00 | 2.92 | | 2.96 | 3.00 | | | 24 | |
| DVD player | 2.92 | 1.02 | 2.46 | 3.39 | 1.00 | 4.67 | 2.90 | | 2.90 | 2.95 | | | 21 | |
| Shoes | 2.92 | 0.95 | 2.49 | 3.35 | 1.67 | 5.33 | 2.86 | | 2.90 | 3.00 | | | 21 | |
| Contact lenses | 2.92 | 0.90 | 2.54 | 3.30 | 1.00 | 4.33 | 2.79 | | 2.83 | 3.13 | | | 24 | |
| Microwave | 2.86 | 0.91 | 2.48 | 3.25 | 1.00 | 5.00 | 2.92 | | 2.88 | 2.79 | | | 24 | |
| Chair | 2.86 | 0.93 | 2.43 | 3.28 | 1.00 | 5.00 | 2.76 | | 2.86 | 2.95 | | | 21 | |
| Power bank | 2.86 | 1.01 | 2.40 | 3.32 | 1.00 | 4.67 | 2.76 | | 2.81 | 3.00 | | | 21 | |
| Vase | 2.83 | 0.94 | 2.44 | 3.23 | 1.00 | 4.33 | 2.92 | | 2.83 | 2.75 | | | 24 | |
| Dress | 2.79 | 0.79 | 2.44 | 3.15 | 2.00 | 4.33 | 2.81 | | 2.76 | 2.81 | | | 21 | |
| Makeup | 2.76 | 1.23 | 2.24 | 3.28 | 1.00 | 4.67 | 2.42 | | 2.92 | 2.96 | | | 24 | |
| Gym subscription | 2.75 | 1.11 | 2.24 | 3.25 | 1.00 | 5.33 | 2.71 | | 2.76 | 2.76 | | | 21 | |
| Massage | 2.75 | 0.93 | 2.32 | 3.17 | 1.00 | 5.00 | 2.71 | | 2.76 | 2.76 | | | 21 | |
| Curtains | 2.73 | 0.96 | 2.29 | 3.17 | 1.00 | 5.00 | 2.67 | | 2.71 | 2.81 | | | 21 | |
| Dinner at a restaurant | 2.70 | 0.84 | 2.31 | 3.08 | 1.67 | 4.67 | 2.76 | | 2.52 | 2.81 | | | 21 | |
| Whiskey | 2.70 | 1.32 | 2.10 | 3.30 | 1.00 | 5.33 | 2.67 | | 2.67 | 2.76 | | | 21 | |
| Baby diapers | 2.69 | 1.24 | 2.17 | 3.22 | 1.00 | 4.67 | 2.38 | | 2.83 | 2.88 | | | 24 | |
| Wine | 2.68 | 0.95 | 2.25 | 3.12 | 1.00 | 4.33 | 2.57 | | 2.71 | 2.76 | | | 21 | |
| Laptop bag | 2.60 | 0.89 | 2.20 | 3.01 | 1.00 | 4.00 | 2.57 | | 2.57 | 2.67 | | | 21 | |
| Blinds | 2.58 | 0.94 | 2.18 | 2.98 | 1.00 | 4.33 | 2.54 | | 2.46 | 2.75 | | | 24 | |
| Backpack | 2.54 | 0.92 | 2.12 | 2.96 | 1.00 | 5.00 | 2.52 | | 2.48 | 2.62 | | | 21 | |
| Dessert at a restaurant | 2.53 | 1.14 | 2.05 | 3.01 | 1.00 | 4.67 | 2.25 | | 2.79 | 2.54 | | | 24 | |
| Cutlery | 2.52 | 0.85 | 2.14 | 2.91 | 1.00 | 3.67 | 2.48 | | 2.52 | 2.57 | | | 21 | |
| Shirt | 2.52 | 1.01 | 2.06 | 2.99 | 1.00 | 4.00 | 2.38 | | 2.57 | 2.62 | | | 21 | |
| Pants | 2.46 | 0.62 | 2.18 | 2.74 | 1.67 | 4.00 | 2.48 | | 2.43 | 2.48 | | | 21 | |
| Entrée at a restaurant | 2.44 | 0.73 | 2.11 | 2.78 | 1.67 | 4.00 | 2.48 | | 2.43 | 2.43 | | | 21 | |
| Museum ticket | 2.43 | 1.01 | 1.97 | 2.89 | 1.00 | 5.00 | 2.43 | | 2.38 | 2.48 | | | 21 | |
| Toaster | 2.40 | 0.91 | 1.98 | 2.81 | 1.00 | 4.00 | 2.33 | | 2.43 | 2.43 | | | 21 | |
| Nature park ticket | 2.40 | 0.83 | 2.02 | 2.77 | 1.00 | 4.00 | 2.38 | | 2.33 | 2.48 | | | 21 | |
| Cigarettes | 2.40 | 1.22 | 1.84 | 2.95 | 1.00 | 6.00 | 2.38 | | 2.48 | 2.33 | | | 21 | |
| Blanket | 2.38 | 0.92 | 1.96 | 2.80 | 1.00 | 4.33 | 2.33 | | 2.43 | 2.38 | | | 21 | |
| Fairground ticket | 2.37 | 1.22 | 1.81 | 2.92 | 1.00 | 6.00 | 2.33 | | 2.33 | 2.43 | | | 21 | |
| Appetizers | 2.32 | 1.18 | 1.82 | 2.82 | 1.00 | 4.67 | 2.21 | | 2.33 | 2.42 | | | 24 | |
| Wall clock | 2.30 | 0.98 | 1.86 | 2.75 | 1.00 | 4.33 | 2.19 | | 2.33 | 2.38 | | | 21 | |
| Online streaming subscription | 2.27 | 0.85 | 1.88 | 2.66 | 1.00 | 4.33 | 2.24 | | 2.29 | 2.29 | | | 21 | |
| Pillow | 2.16 | 0.92 | 1.74 | 2.58 | 1.00 | 4.00 | 2.10 | | 2.10 | 2.29 | | | 21 | |
| Insecticide | 2.14 | 1.02 | 1.71 | 2.57 | 1.00 | 4.33 | 2.25 | | 2.04 | 2.13 | | | 24 | |
| Pajamas | 2.13 | 0.96 | 1.69 | 2.57 | 1.00 | 4.33 | 2.14 | | 2.14 | 2.10 | | | 21 | |
| Movie ticket | 2.13 | 0.79 | 1.77 | 2.49 | 1.00 | 4.00 | 2.10 | | 2.10 | 2.19 | | | 21 | |
| Book | 2.13 | 1.12 | 1.62 | 2.64 | 1.00 | 4.67 | 2.14 | | 2.14 | 2.10 | | | 21 | |
| T-shirt | 2.13 | 1.04 | 1.65 | 2.60 | 1.00 | 4.00 | 1.95 | | 2.10 | 2.33 | | | 21 | |
| Umbrella | 2.11 | 1.06 | 1.66 | 2.56 | 1.00 | 4.33 | 2.17 | | 2.04 | 2.13 | | | 24 | |
| Plants | 2.11 | 0.93 | 1.69 | 2.54 | 1.00 | 4.00 | 2.10 | | 2.05 | 2.19 | | | 21 | |
| Calculator | 2.10 | 0.80 | 1.76 | 2.43 | 1.00 | 3.67 | 1.88 | | 2.21 | 2.21 | | | 24 | |
| CD | 2.10 | 1.05 | 1.62 | 2.57 | 1.00 | 4.00 | 1.95 | | 2.14 | 2.19 | | | 21 | |
| Water bottle | 2.06 | 1.32 | 1.50 | 2.61 | 1.00 | 4.67 | 1.79 | | 2.17 | 2.21 | | | 24 | |
| After shave | 2.04 | 1.23 | 1.52 | 2.56 | 1.00 | 5.33 | 1.88 | | 2.13 | 2.13 | | | 24 | |
| Laundry detergent | 2.04 | 1.03 | 1.61 | 2.48 | 1.00 | 4.67 | 1.88 | | 2.13 | 2.13 | | | 24 | |
| Towel | 2.02 | 0.89 | 1.61 | 2.42 | 1.00 | 4.00 | 2.00 | | 2.00 | 2.05 | | | 21 | |
| Picture frame | 2.02 | 1.11 | 1.51 | 2.52 | 1.00 | 5.00 | 2.05 | | 2.00 | 2.00 | | | 21 | |
| Razor blades | 1.98 | 0.79 | 1.62 | 2.34 | 1.00 | 3.00 | 2.00 | | 2.00 | 1.95 | | | 21 | |
| DVD | 1.98 | 0.97 | 1.54 | 2.43 | 1.00 | 4.00 | 1.95 | | 1.86 | 2.14 | | | 21 | |
| Pizza | 1.98 | 0.91 | 1.57 | 2.40 | 1.00 | 4.33 | 2.00 | | 2.05 | 1.90 | | | 21 | |
| Underwear | 1.95 | 0.85 | 1.56 | 2.34 | 1.00 | 4.33 | 1.95 | | 1.95 | 1.95 | | | 21 | |
| Skin moisturizer | 1.95 | 1.07 | 1.47 | 2.44 | 1.00 | 4.33 | 1.90 | | 1.95 | 2.00 | | | 21 | |
| **Dishwashing brush** | 1.94 | 1.07 | 1.68 | 2.21 | 1.00 | 6.00 | 1.89 | | 1.92 | 2.02 | | | 66 | |
| Beer | 1.94 | 0.87 | 1.54 | 2.33 | 1.00 | 4.00 | 1.86 | | 1.95 | 2.00 | | | 21 | |
| USB flash drive | 1.92 | 0.69 | 1.61 | 2.23 | 1.00 | 3.67 | 1.95 | | 1.86 | 1.95 | | | 21 | |
| Trash bin | 1.92 | 1.09 | 1.46 | 2.38 | 1.00 | 4.33 | 1.79 | | 1.96 | 2.00 | | | 24 | |
| Milk | 1.92 | 1.33 | 1.36 | 2.48 | 1.00 | 5.00 | 1.75 | | 2.04 | 1.96 | | | 24 | |
| Flashlight | 1.90 | 0.93 | 1.48 | 2.33 | 1.00 | 4.67 | 1.90 | | 1.90 | 1.90 | | | 21 | |
| Oven mitts | 1.90 | 1.08 | 1.45 | 2.36 | 1.00 | 4.33 | 1.83 | | 1.88 | 2.00 | | | 24 | |
| **Pen** | 1.89 | 1.14 | 1.61 | 2.17 | 1.00 | 5.33 | 1.82 | | 1.91 | 1.95 | | | 66 | |
| Ice cream | 1.89 | 1.24 | 1.37 | 2.41 | 1.00 | 4.67 | 1.83 | | 1.92 | 1.92 | | | 24 | |
| Ceramic mug | 1.89 | 1.20 | 1.38 | 2.40 | 1.00 | 5.00 | 1.75 | | 2.00 | 1.92 | | | 24 | |
| Peanut butter | 1.86 | 1.02 | 1.43 | 2.29 | 1.00 | 4.00 | 1.83 | | 1.83 | 1.92 | | | 24 | |
| Glasses (drinking) | 1.86 | 0.99 | 1.41 | 2.31 | 1.00 | 4.00 | 1.71 | | 1.76 | 2.10 | | | 21 | |
| Cookies | 1.85 | 1.27 | 1.31 | 2.39 | 1.00 | 4.67 | 1.71 | | 1.96 | 1.88 | | | 24 | |
| Tea | 1.85 | 1.13 | 1.37 | 2.33 | 1.00 | 4.67 | 1.83 | | 1.83 | 1.88 | | | 24 | |
| Soft drink | 1.83 | 1.22 | 1.32 | 2.35 | 1.00 | 4.67 | 1.63 | | 2.00 | 1.88 | | | 24 | |
| Shower gel | 1.81 | 1.08 | 1.32 | 2.30 | 1.00 | 4.00 | 1.71 | | 1.81 | 1.90 | | | 21 | |
| Coffee | 1.79 | 0.96 | 1.36 | 2.23 | 1.00 | 4.33 | 1.71 | | 1.81 | 1.86 | | | 21 | |
| Notebook | 1.78 | 0.90 | 1.40 | 2.16 | 1.00 | 4.00 | 1.83 | | 1.75 | 1.75 | | | 24 | |
| Sunscreen | 1.78 | 0.86 | 1.39 | 2.17 | 1.00 | 4.33 | 1.67 | | 1.81 | 1.86 | | | 21 | |
| Vegetables | 1.78 | 1.21 | 1.23 | 2.33 | 1.00 | 4.67 | 1.71 | | 1.76 | 1.86 | | | 21 | |
| Computer mouse | 1.76 | 0.78 | 1.41 | 2.11 | 1.00 | 3.67 | 1.71 | | 1.76 | 1.81 | | | 21 | |
| Aspirin | 1.72 | 1.18 | 1.22 | 2.22 | 1.00 | 5.00 | 1.54 | | 1.79 | 1.83 | | | 24 | |
| Shampoo | 1.65 | 0.79 | 1.29 | 2.01 | 1.00 | 4.33 | 1.62 | | 1.62 | 1.71 | | | 21 | |
| Coasters | 1.65 | 0.87 | 1.26 | 2.04 | 1.00 | 4.00 | 1.62 | | 1.62 | 1.71 | | | 21 | |
| Deodorant | 1.63 | 0.96 | 1.22 | 2.03 | 1.00 | 4.33 | 1.71 | | 1.50 | 1.67 | | | 24 | |
| Fruit | 1.62 | 0.85 | 1.23 | 2.01 | 1.00 | 4.33 | 1.62 | | 1.67 | 1.57 | | | 21 | |
| Mouthwash | 1.62 | 0.79 | 1.26 | 1.98 | 1.00 | 4.33 | 1.62 | | 1.57 | 1.67 | | | 21 | |
| Toothpaste | 1.60 | 1.08 | 1.11 | 2.10 | 1.00 | 4.00 | 1.48 | | 1.62 | 1.71 | | | 21 | |
| Eggs | 1.59 | 0.91 | 1.17 | 2.00 | 1.00 | 4.00 | 1.52 | | 1.67 | 1.57 | | | 21 | |
| Toilet paper | 1.57 | 1.12 | 1.06 | 2.08 | 1.00 | 4.67 | 1.48 | | 1.57 | 1.67 | | | 21 | |
| Hangers | 1.57 | 0.97 | 1.13 | 2.01 | 1.00 | 4.00 | 1.57 | | 1.52 | 1.62 | | | 21 | |
| Scissors | 1.56 | 0.73 | 1.22 | 1.89 | 1.00 | 3.00 | 1.62 | | 1.48 | 1.57 | | | 21 | |
| Cooking oil | 1.52 | 0.86 | 1.13 | 1.92 | 1.00 | 4.67 | 1.48 | | 1.52 | 1.57 | | | 21 | |
| Toilet brush | 1.52 | 0.69 | 1.21 | 1.84 | 1.00 | 3.67 | 1.57 | | 1.48 | 1.52 | | | 21 | |
| Trash bag | 1.52 | 0.69 | 1.21 | 1.84 | 1.00 | 3.33 | 1.57 | | 1.57 | 1.43 | | | 21 | |
| Disposable plastic cups | 1.49 | 0.98 | 1.05 | 1.94 | 1.00 | 4.00 | 1.38 | | 1.52 | 1.57 | | | 21 | |
| Adhesive bandage | 1.46 | 0.80 | 1.10 | 1.82 | 1.00 | 4.33 | 1.52 | | 1.43 | 1.43 | | | 21 | |
| Dental floss | 1.46 | 0.97 | 1.02 | 1.90 | 1.00 | 4.00 | 1.38 | | 1.48 | 1.52 | | | 21 | |
| Breakfast cereal | 1.46 | 0.70 | 1.14 | 1.78 | 1.00 | 3.67 | 1.52 | | 1.43 | 1.43 | | | 21 | |
| Cleaning cloth | 1.44 | 0.74 | 1.11 | 1.78 | 1.00 | 4.00 | 1.43 | | 1.48 | 1.43 | | | 21 | |
| Chocolate bar | 1.44 | 0.85 | 1.06 | 1.83 | 1.00 | 3.67 | 1.38 | | 1.43 | 1.52 | | | 21 | |
| **Paper clips** | 1.44 | 0.91 | 1.22 | 1.66 | 1.00 | 4.67 | 1.41 | | 1.44 | 1.47 | | | 66 | |
| Potato chips | 1.43 | 0.75 | 1.09 | 1.77 | 1.00 | 3.67 | 1.43 | | 1.43 | 1.43 | | | 21 | |
| Dish detergent | 1.43 | 0.63 | 1.14 | 1.72 | 1.00 | 3.33 | 1.38 | | 1.43 | 1.48 | | | 21 | |
| Bread | 1.43 | 0.68 | 1.12 | 1.74 | 1.00 | 3.67 | 1.33 | | 1.52 | 1.43 | | | 21 | |
| Postcard | 1.40 | 1.00 | 0.94 | 1.85 | 1.00 | 4.00 | 1.33 | | 1.38 | 1.48 | | | 21 | |
| Yogurt | 1.33 | 0.73 | 1.00 | 1.67 | 1.00 | 3.00 | 1.29 | | 1.29 | 1.43 | | | 21 | |
| Soap | 1.32 | 0.67 | 1.01 | 1.62 | 1.00 | 3.67 | 1.38 | | 1.29 | 1.29 | | | 21 | |
| Air freshener | 1.32 | 0.67 | 1.01 | 1.62 | 1.00 | 3.67 | 1.29 | | 1.29 | 1.38 | | | 21 | |
| Chewing gum | 1.25 | 0.70 | 0.94 | 1.57 | 1.00 | 4.00 | 1.33 | | 1.19 | 1.24 | | | 21 | |

Table 6. Descriptive statistics for product’s attitude (ordered in descending order).

|  |  |  |  |  |  |  |  | **Items** | | |  |  | |  |
| --- | --- | --- | --- | --- | --- | --- | --- | --- | --- | --- | --- | --- | --- | --- |
| **Product** | **Mean (across 3 items)** | **SD** | **CI -95** | **CI 95** | **Min** | **Max** | **1. How positive do you feel about this product?** | | **2. How negative do you feel about this product? (reverse-coded)** | **3. How much do you like this product?** | | | **n** | |
| Television | 5.36 | 0.88 | 4.97 | 5.76 | 3.33 | 6.00 | 5.23 | | 1.50 | 5.36 | | | 22 | |
| Pillow | 5.30 | 0.83 | 4.94 | 5.67 | 3.67 | 6.00 | 5.14 | | 1.41 | 5.18 | | | 22 | |
| Soap | 5.25 | 0.94 | 4.83 | 5.68 | 3.00 | 6.00 | 5.29 | | 1.57 | 5.05 | | | 21 | |
| Desktop computer | 5.25 | 0.79 | 4.90 | 5.59 | 3.67 | 6.00 | 5.30 | | 2.00 | 5.43 | | | 23 | |
| Air conditioner | 5.24 | 0.96 | 4.80 | 5.68 | 2.67 | 6.00 | 5.33 | | 1.81 | 5.19 | | | 21 | |
| Plants | 5.23 | 0.92 | 4.83 | 5.63 | 3.00 | 6.00 | 5.43 | | 1.61 | 4.87 | | | 23 | |
| Pizza | 5.23 | 1.02 | 4.78 | 5.68 | 2.33 | 6.00 | 5.18 | | 1.59 | 5.09 | | | 22 | |
| House | 5.23 | 0.88 | 4.84 | 5.62 | 3.67 | 6.00 | 5.09 | | 1.36 | 4.95 | | | 22 | |
| Chocolate bar | 5.21 | 0.88 | 4.81 | 5.61 | 2.33 | 6.00 | 5.24 | | 1.95 | 5.33 | | | 21 | |
| Piano | 5.21 | 0.80 | 4.84 | 5.57 | 3.67 | 6.00 | 5.43 | | 1.52 | 4.71 | | | 21 | |
| Laptop | 5.16 | 0.90 | 4.77 | 5.55 | 3.67 | 6.00 | 5.17 | | 2.00 | 5.30 | | | 23 | |
| Fruit | 5.16 | 0.91 | 4.74 | 5.57 | 3.00 | 6.00 | 5.19 | | 1.62 | 4.90 | | | 21 | |
| Sun glasses | 5.16 | 0.76 | 4.81 | 5.50 | 3.33 | 6.00 | 5.10 | | 1.57 | 4.95 | | | 21 | |
| Camera | 5.15 | 1.04 | 4.69 | 5.61 | 3.33 | 6.00 | 5.09 | | 1.64 | 5.00 | | | 22 | |
| Mattress | 5.14 | 0.84 | 4.78 | 5.51 | 3.67 | 6.00 | 5.09 | | 1.87 | 5.22 | | | 23 | |
| Shirt | 5.14 | 0.73 | 4.81 | 5.48 | 4.00 | 6.00 | 4.95 | | 1.48 | 4.95 | | | 21 | |
| Car | 5.14 | 0.77 | 4.79 | 5.49 | 3.67 | 6.00 | 5.24 | | 1.71 | 4.90 | | | 21 | |
| Towel | 5.13 | 0.85 | 4.74 | 5.51 | 3.33 | 6.00 | 5.05 | | 1.71 | 5.05 | | | 21 | |
| **Ice cream** | 5.12 | 1.09 | 4.85 | 5.38 | 1.67 | 6.00 | 5.18 | | 1.97 | 5.14 | | | 66 | |
| Eggs | 5.11 | 0.99 | 4.67 | 5.54 | 2.67 | 6.00 | 5.09 | | 1.73 | 4.95 | | | 22 | |
| Couch | 5.10 | 0.97 | 4.65 | 5.54 | 2.67 | 6.00 | 5.05 | | 1.71 | 4.95 | | | 21 | |
| Bedding | 5.08 | 0.82 | 4.71 | 5.45 | 3.67 | 6.00 | 5.10 | | 1.62 | 4.76 | | | 21 | |
| Blanket | 5.07 | 1.00 | 4.64 | 5.50 | 3.67 | 6.00 | 5.04 | | 2.00 | 5.17 | | | 23 | |
| Movie ticket | 5.06 | 0.73 | 4.73 | 5.40 | 4.00 | 6.00 | 5.00 | | 1.90 | 5.10 | | | 21 | |
| Book | 5.06 | 1.16 | 4.55 | 5.58 | 1.67 | 6.00 | 5.09 | | 1.86 | 4.95 | | | 22 | |
| Dessert at a restaurant | 5.05 | 1.31 | 4.47 | 5.62 | 2.00 | 6.00 | 5.18 | | 1.95 | 4.91 | | | 22 | |
| Dinner at a restaurant | 5.05 | 1.09 | 4.56 | 5.53 | 2.33 | 6.00 | 5.05 | | 1.77 | 4.86 | | | 22 | |
| T-shirt | 5.03 | 0.85 | 4.65 | 5.41 | 3.33 | 6.00 | 4.91 | | 1.59 | 4.77 | | | 22 | |
| Shoes | 5.02 | 0.95 | 4.58 | 5.45 | 2.67 | 6.00 | 5.10 | | 2.00 | 4.95 | | | 21 | |
| Tea | 5.02 | 0.88 | 4.63 | 5.40 | 3.67 | 6.00 | 4.77 | | 1.50 | 4.77 | | | 22 | |
| Underwear | 5.00 | 0.87 | 4.62 | 5.38 | 3.33 | 6.00 | 4.59 | | 1.55 | 4.95 | | | 22 | |
| Pants | 5.00 | 1.03 | 4.54 | 5.46 | 2.67 | 6.00 | 4.68 | | 1.50 | 4.82 | | | 22 | |
| Entrée at a restaurant | 4.99 | 1.02 | 4.55 | 5.43 | 3.33 | 6.00 | 5.09 | | 2.13 | 5.00 | | | 23 | |
| Pajamas | 4.98 | 0.90 | 4.59 | 5.38 | 3.33 | 6.00 | 4.77 | | 1.59 | 4.77 | | | 22 | |
| DVD player | 4.98 | 1.03 | 4.52 | 5.45 | 2.00 | 6.00 | 5.00 | | 1.86 | 4.81 | | | 21 | |
| Appetizers | 4.95 | 1.15 | 4.44 | 5.47 | 2.00 | 6.00 | 4.68 | | 1.73 | 4.91 | | | 22 | |
| Tablet | 4.95 | 1.12 | 4.44 | 5.46 | 1.67 | 6.00 | 5.10 | | 2.10 | 4.86 | | | 21 | |
| Cookies | 4.94 | 1.11 | 4.46 | 5.42 | 2.00 | 6.00 | 5.00 | | 2.22 | 5.04 | | | 23 | |
| USB flash drive | 4.94 | 0.81 | 4.57 | 5.30 | 4.00 | 6.00 | 5.10 | | 1.95 | 4.67 | | | 21 | |
| Smartphone | 4.94 | 1.10 | 4.43 | 5.44 | 2.33 | 6.00 | 4.90 | | 2.05 | 4.95 | | | 21 | |
| Boots | 4.93 | 0.96 | 4.51 | 5.34 | 3.33 | 6.00 | 4.83 | | 2.00 | 4.96 | | | 23 | |
| Toothpaste | 4.93 | 0.86 | 4.55 | 5.30 | 3.33 | 6.00 | 4.91 | | 1.91 | 4.78 | | | 23 | |
| Shampoo | 4.92 | 0.94 | 4.51 | 5.34 | 3.33 | 6.00 | 4.64 | | 1.64 | 4.77 | | | 22 | |
| Museum ticket | 4.92 | 1.14 | 4.42 | 5.43 | 2.00 | 6.00 | 4.77 | | 1.73 | 4.73 | | | 22 | |
| Yogurt | 4.92 | 1.10 | 4.44 | 5.41 | 2.67 | 6.00 | 4.86 | | 1.73 | 4.64 | | | 22 | |
| Toilet paper | 4.91 | 0.93 | 4.50 | 5.32 | 3.33 | 6.00 | 4.50 | | 1.41 | 4.64 | | | 22 | |
| Toaster | 4.90 | 0.93 | 4.48 | 5.33 | 3.00 | 6.00 | 5.00 | | 1.95 | 4.67 | | | 21 | |
| **Vacation package** | 4.90 | 1.07 | 4.63 | 5.16 | 1.67 | 6.00 | 4.88 | | 2.14 | 4.95 | | | 66 | |
| Gas outdoor grill | 4.89 | 1.08 | 4.41 | 5.37 | 2.33 | 6.00 | 4.55 | | 1.77 | 4.91 | | | 22 | |
| 3D printer | 4.89 | 0.97 | 4.46 | 5.33 | 2.67 | 6.00 | 4.86 | | 1.64 | 4.45 | | | 22 | |
| Printer | 4.89 | 0.80 | 4.53 | 5.25 | 3.67 | 6.00 | 5.05 | | 2.10 | 4.71 | | | 21 | |
| GPS | 4.89 | 0.88 | 4.49 | 5.29 | 3.33 | 6.00 | 4.90 | | 2.00 | 4.76 | | | 21 | |
| Table | 4.88 | 0.79 | 4.53 | 5.23 | 3.33 | 6.00 | 4.55 | | 1.55 | 4.64 | | | 22 | |
| Flashlight | 4.87 | 0.90 | 4.48 | 5.26 | 3.33 | 6.00 | 4.74 | | 1.91 | 4.78 | | | 23 | |
| Radio | 4.86 | 1.08 | 4.38 | 5.34 | 2.00 | 6.00 | 4.73 | | 1.86 | 4.73 | | | 22 | |
| Vacuum cleaner | 4.86 | 0.82 | 4.50 | 5.23 | 3.33 | 6.00 | 4.64 | | 1.73 | 4.68 | | | 22 | |
| Breakfast cereal | 4.83 | 0.73 | 4.49 | 5.16 | 4.00 | 6.00 | 4.81 | | 1.81 | 4.48 | | | 21 | |
| Blinds | 4.82 | 0.96 | 4.39 | 5.24 | 3.00 | 6.00 | 4.59 | | 1.77 | 4.64 | | | 22 | |
| Eyeglasses | 4.82 | 1.23 | 4.27 | 5.36 | 2.00 | 6.00 | 4.55 | | 1.73 | 4.64 | | | 22 | |
| Computer mouse | 4.81 | 1.02 | 4.37 | 5.25 | 2.33 | 6.00 | 4.65 | | 2.09 | 4.87 | | | 23 | |
| Glasses (drinking) | 4.81 | 0.77 | 4.48 | 5.14 | 3.67 | 6.00 | 4.87 | | 2.13 | 4.70 | | | 23 | |
| Chair | 4.81 | 1.01 | 4.38 | 5.25 | 3.00 | 6.00 | 4.70 | | 2.04 | 4.78 | | | 23 | |
| Microwave | 4.81 | 1.12 | 4.30 | 5.32 | 2.33 | 6.00 | 4.95 | | 2.24 | 4.71 | | | 21 | |
| CD | 4.81 | 0.79 | 4.45 | 5.17 | 3.67 | 6.00 | 4.71 | | 1.81 | 4.52 | | | 21 | |
| Laptop bag | 4.80 | 0.76 | 4.47 | 5.14 | 3.67 | 6.00 | 4.55 | | 1.59 | 4.45 | | | 22 | |
| **Massage** | 4.80 | 1.16 | 4.51 | 5.08 | 1.00 | 6.00 | 4.91 | | 2.20 | 4.68 | | | 66 | |
| Coffee | 4.80 | 1.28 | 4.24 | 5.35 | 2.00 | 6.00 | 4.83 | | 2.39 | 4.96 | | | 23 | |
| Bicycle | 4.79 | 1.10 | 4.29 | 5.30 | 1.67 | 6.00 | 4.86 | | 1.90 | 4.43 | | | 21 | |
| Cutlery | 4.78 | 0.87 | 4.40 | 5.16 | 3.33 | 6.00 | 4.78 | | 2.04 | 4.61 | | | 23 | |
| Backpack | 4.77 | 0.92 | 4.36 | 5.18 | 3.00 | 6.00 | 4.45 | | 1.55 | 4.41 | | | 22 | |
| Vegetables | 4.77 | 1.07 | 4.30 | 5.25 | 2.67 | 6.00 | 4.68 | | 1.77 | 4.41 | | | 22 | |
| Desk | 4.77 | 0.85 | 4.40 | 5.15 | 3.00 | 6.00 | 4.41 | | 1.77 | 4.68 | | | 22 | |
| Speakers | 4.77 | 1.06 | 4.31 | 5.23 | 3.00 | 6.00 | 4.78 | | 2.30 | 4.83 | | | 23 | |
| Deodorant | 4.76 | 1.05 | 4.28 | 5.24 | 2.67 | 6.00 | 4.71 | | 2.00 | 4.57 | | | 21 | |
| Wine | 4.76 | 1.50 | 4.09 | 5.42 | 1.67 | 6.00 | 4.64 | | 1.91 | 4.55 | | | 22 | |
| Pen | 4.75 | 0.87 | 4.38 | 5.13 | 3.33 | 6.00 | 4.70 | | 2.09 | 4.65 | | | 23 | |
| Painting | 4.75 | 0.86 | 4.38 | 5.13 | 3.33 | 6.00 | 4.52 | | 1.83 | 4.57 | | | 23 | |
| Concert ticket | 4.74 | 1.55 | 4.05 | 5.43 | 1.00 | 6.00 | 4.68 | | 2.05 | 4.59 | | | 22 | |
| Laundry detergent | 4.74 | 0.91 | 4.35 | 5.13 | 3.33 | 6.00 | 4.74 | | 2.30 | 4.78 | | | 23 | |
| Nature park ticket | 4.73 | 1.40 | 4.09 | 5.37 | 1.00 | 6.00 | 4.71 | | 2.05 | 4.52 | | | 21 | |
| Headphones | 4.72 | 1.28 | 4.17 | 5.28 | 1.00 | 6.00 | 4.74 | | 2.22 | 4.65 | | | 23 | |
| Oven mitts | 4.72 | 1.00 | 4.29 | 5.16 | 3.00 | 6.00 | 4.65 | | 2.13 | 4.65 | | | 23 | |
| Bread | 4.72 | 1.32 | 4.16 | 5.29 | 1.00 | 6.00 | 4.74 | | 2.35 | 4.78 | | | 23 | |
| Sunscreen | 4.70 | 1.38 | 4.07 | 5.33 | 1.00 | 6.00 | 4.81 | | 2.24 | 4.52 | | | 21 | |
| Umbrella | 4.70 | 0.95 | 4.29 | 5.11 | 3.00 | 6.00 | 4.70 | | 2.13 | 4.52 | | | 23 | |
| Mouthwash | 4.70 | 1.08 | 4.23 | 5.16 | 2.33 | 6.00 | 4.74 | | 2.22 | 4.57 | | | 23 | |
| Calculator | 4.68 | 0.97 | 4.24 | 5.13 | 3.33 | 6.00 | 4.95 | | 2.19 | 4.29 | | | 21 | |
| Potato chips | 4.68 | 1.04 | 4.22 | 5.14 | 3.00 | 6.00 | 4.45 | | 1.95 | 4.55 | | | 22 | |
| Scissors | 4.68 | 0.96 | 4.26 | 5.11 | 2.67 | 6.00 | 4.18 | | 1.68 | 4.55 | | | 22 | |
| Postcard | 4.65 | 0.70 | 4.33 | 4.97 | 3.33 | 6.00 | 4.52 | | 1.76 | 4.19 | | | 21 | |
| Online streaming subscription | 4.64 | 1.41 | 4.03 | 5.25 | 1.00 | 6.00 | 4.65 | | 2.48 | 4.74 | | | 23 | |
| Power bank | 4.63 | 0.99 | 4.18 | 5.09 | 2.00 | 6.00 | 4.33 | | 1.71 | 4.29 | | | 21 | |
| Hotel room | 4.63 | 0.87 | 4.24 | 5.03 | 3.00 | 6.00 | 4.67 | | 2.43 | 4.67 | | | 21 | |
| Picture frame | 4.63 | 0.65 | 4.34 | 4.93 | 3.33 | 6.00 | 4.57 | | 2.00 | 4.33 | | | 21 | |
| Watch | 4.62 | 1.42 | 3.99 | 5.25 | 1.67 | 6.00 | 4.50 | | 1.86 | 4.23 | | | 22 | |
| Peanut butter | 4.61 | 1.28 | 4.05 | 5.16 | 1.00 | 6.00 | 4.70 | | 2.65 | 4.78 | | | 23 | |
| Internet provider | 4.61 | 1.28 | 4.04 | 5.17 | 1.00 | 6.00 | 4.64 | | 2.27 | 4.45 | | | 22 | |
| Dish detergent | 4.60 | 0.89 | 4.20 | 5.01 | 3.00 | 6.00 | 4.57 | | 2.19 | 4.43 | | | 21 | |
| Milk | 4.60 | 1.21 | 4.05 | 5.16 | 1.00 | 6.00 | 4.62 | | 2.19 | 4.38 | | | 21 | |
| Ceramic mug | 4.59 | 0.97 | 4.17 | 5.02 | 3.00 | 6.00 | 4.48 | | 2.13 | 4.43 | | | 23 | |
| Shower gel | 4.59 | 1.21 | 4.04 | 5.14 | 1.67 | 6.00 | 4.62 | | 2.05 | 4.19 | | | 21 | |
| Hybrid/Convertible laptop | 4.59 | 1.27 | 4.01 | 5.16 | 1.00 | 6.00 | 4.62 | | 2.24 | 4.38 | | | 21 | |
| Wall clock | 4.58 | 1.04 | 4.11 | 5.04 | 1.67 | 6.00 | 4.32 | | 1.77 | 4.18 | | | 22 | |
| Contact lenses | 4.56 | 1.36 | 3.96 | 5.16 | 1.00 | 6.00 | 4.32 | | 2.05 | 4.41 | | | 22 | |
| Dental floss | 4.56 | 1.01 | 4.09 | 5.02 | 2.67 | 6.00 | 4.52 | | 2.14 | 4.29 | | | 21 | |
| Skin moisturizer | 4.55 | 1.13 | 4.06 | 5.04 | 2.00 | 6.00 | 4.57 | | 2.39 | 4.48 | | | 23 | |
| Guitar | 4.55 | 1.44 | 3.93 | 5.18 | 1.00 | 6.00 | 4.61 | | 2.39 | 4.43 | | | 23 | |
| Bracelet | 4.55 | 1.10 | 4.06 | 5.03 | 2.00 | 6.00 | 4.27 | | 1.68 | 4.05 | | | 22 | |
| Plane ticket | 4.55 | 1.40 | 3.93 | 5.17 | 1.00 | 6.00 | 4.32 | | 2.09 | 4.41 | | | 22 | |
| Notebook | 4.54 | 1.18 | 4.03 | 5.05 | 1.00 | 6.00 | 4.48 | | 2.22 | 4.35 | | | 23 | |
| Air freshener | 4.54 | 1.22 | 4.01 | 5.06 | 2.00 | 6.00 | 4.35 | | 2.35 | 4.61 | | | 23 | |
| Coasters | 4.51 | 0.84 | 4.13 | 4.89 | 2.67 | 6.00 | 4.24 | | 2.00 | 4.29 | | | 21 | |
| Fairground ticket | 4.50 | 1.19 | 3.97 | 5.03 | 2.33 | 6.00 | 4.36 | | 1.95 | 4.09 | | | 22 | |
| PC game | 4.45 | 1.56 | 3.76 | 5.15 | 1.00 | 6.00 | 4.36 | | 2.27 | 4.27 | | | 22 | |
| Dress | 4.45 | 1.26 | 3.90 | 4.99 | 1.00 | 6.00 | 4.26 | | 2.13 | 4.22 | | | 23 | |
| Vase | 4.44 | 0.92 | 4.03 | 4.85 | 1.67 | 6.00 | 4.05 | | 1.86 | 4.14 | | | 22 | |
| Cooking oil | 4.41 | 1.23 | 3.85 | 4.97 | 1.67 | 6.00 | 4.29 | | 2.43 | 4.38 | | | 21 | |
| Curtains | 4.41 | 1.03 | 3.96 | 4.85 | 2.33 | 6.00 | 4.30 | | 2.35 | 4.26 | | | 23 | |
| Trash bag | 4.38 | 1.12 | 3.87 | 4.89 | 1.67 | 6.00 | 4.29 | | 2.38 | 4.24 | | | 21 | |
| Water bottle | 4.37 | 1.27 | 3.79 | 4.94 | 1.00 | 6.00 | 4.38 | | 2.67 | 4.38 | | | 21 | |
| Apartment | 4.36 | 1.02 | 3.92 | 4.81 | 2.00 | 6.00 | 4.43 | | 2.78 | 4.43 | | | 23 | |
| Chewing gum | 4.35 | 1.35 | 3.75 | 4.95 | 2.00 | 6.00 | 4.23 | | 2.36 | 4.18 | | | 22 | |
| Gym subscription | 4.35 | 1.37 | 3.74 | 4.96 | 1.00 | 6.00 | 4.18 | | 2.41 | 4.27 | | | 22 | |
| Cruise trip | 4.32 | 1.53 | 3.66 | 4.98 | 1.00 | 6.00 | 4.52 | | 2.83 | 4.26 | | | 23 | |
| Paper clips | 4.32 | 0.94 | 3.89 | 4.75 | 2.67 | 6.00 | 4.14 | | 2.14 | 3.95 | | | 21 | |
| Medical treatment | 4.32 | 0.90 | 3.91 | 4.73 | 3.33 | 6.00 | 4.48 | | 2.57 | 4.05 | | | 21 | |
| Cleaning cloth | 4.30 | 1.01 | 3.87 | 4.74 | 1.00 | 6.00 | 4.22 | | 2.43 | 4.13 | | | 23 | |
| Aspirin | 4.29 | 1.03 | 3.84 | 4.74 | 1.67 | 6.00 | 4.17 | | 2.43 | 4.13 | | | 23 | |
| Hangers | 4.28 | 1.03 | 3.83 | 4.72 | 2.00 | 6.00 | 4.04 | | 2.30 | 4.13 | | | 23 | |
| Necklace | 4.28 | 1.26 | 3.73 | 4.82 | 1.00 | 6.00 | 4.17 | | 2.48 | 4.13 | | | 23 | |
| After shave | 4.26 | 1.39 | 3.64 | 4.87 | 1.00 | 6.00 | 3.95 | | 2.14 | 3.95 | | | 22 | |
| Trash bin | 4.25 | 1.01 | 3.80 | 4.71 | 2.67 | 6.00 | 4.19 | | 2.33 | 3.90 | | | 21 | |
| Razor blades | 4.22 | 0.91 | 3.82 | 4.61 | 2.00 | 6.00 | 4.17 | | 2.57 | 4.04 | | | 23 | |
| Suit | 4.16 | 1.36 | 3.57 | 4.75 | 1.00 | 6.00 | 4.30 | | 2.74 | 3.91 | | | 23 | |
| Baby diapers | 4.12 | 1.56 | 3.43 | 4.81 | 1.00 | 6.00 | 3.86 | | 2.50 | 4.00 | | | 22 | |
| Adhesive bandage | 4.12 | 1.27 | 3.57 | 4.67 | 1.00 | 6.00 | 3.96 | | 2.52 | 3.91 | | | 23 | |
| DVD | 4.09 | 1.40 | 3.48 | 4.69 | 1.00 | 6.00 | 3.96 | | 2.74 | 4.04 | | | 23 | |
| Beer | 4.06 | 1.79 | 3.25 | 4.88 | 1.00 | 6.00 | 4.00 | | 2.62 | 3.81 | | | 21 | |
| Room (renting) | 4.06 | 1.14 | 3.54 | 4.58 | 1.00 | 6.00 | 4.00 | | 2.52 | 3.71 | | | 21 | |
| Life insurance | 4.06 | 1.06 | 3.60 | 4.52 | 1.00 | 6.00 | 4.09 | | 2.83 | 3.91 | | | 23 | |
| Makeup | 4.02 | 1.50 | 3.33 | 4.70 | 1.00 | 6.00 | 4.00 | | 2.71 | 3.76 | | | 21 | |
| Perfume | 3.86 | 1.53 | 3.19 | 4.52 | 1.00 | 6.00 | 3.65 | | 3.09 | 4.00 | | | 23 | |
| **Toilet brush** | 3.81 | 1.03 | 3.55 | 4.06 | 1.00 | 6.00 | 3.53 | | 2.82 | 3.71 | | | 66 | |
| Whiskey | 3.76 | 1.77 | 2.97 | 4.54 | 1.00 | 6.00 | 3.64 | | 2.82 | 3.45 | | | 22 | |
| Dishwashing brush | 3.75 | 1.09 | 3.28 | 4.23 | 1.00 | 5.33 | 3.57 | | 2.74 | 3.43 | | | 23 | |
| Motorcycle | 3.57 | 1.41 | 2.93 | 4.21 | 1.00 | 6.00 | 3.57 | | 3.43 | 3.57 | | | 21 | |
| Soft drink | 3.49 | 1.52 | 2.83 | 4.15 | 1.00 | 6.00 | 3.22 | | 3.26 | 3.52 | | | 23 | |
| Disposable plastic cups | 3.35 | 1.48 | 2.71 | 3.99 | 1.00 | 6.00 | 3.22 | | 3.43 | 3.26 | | | 23 | |
| **Insecticide** | 3.22 | 1.40 | 2.87 | 3.56 | 1.00 | 6.00 | 3.03 | | 3.52 | 3.14 | | | 66 | |
| **Cigarettes** | 2.07 | 1.41 | 1.72 | 2.41 | 1.00 | 6.00 | 2.02 | | 5.02 | 2.20 | | | 66 | |

**Note:** calibration products in bold.
