# Supplementary material for: Norms for 150 consumer products: Perceived complexity, quality objectivity, material/experiential nature, perceived price, familiarity and attitude
Source: PLoS One. 2020 Sep 21;15(9):e0238848. doi: 10.1371/journal.pone.0238848 (PMC7505470; doi:10.1371/journal.pone.0238848)
Supplement: Attachment — Submitted filename: Response to reviewers.docx [file pone.0238848.s002.docx]

**RESPONSE LETTER**

Filipe Loureiro

William James Center for Research

ISPA - Instituto Universitário

Lisbon, 1149-041

Monday, April 27^th^, 2020

Dr. Claudio Soregaroli

Academic Editor

PLOS ONE

Dear Dr. Claudio Soregaroli,

First of all, we would like to thank you and the reviewers for the review process regarding our submission of the paper entitled *“Norms for 150 consumer products: perceived complexity, quality objectivity, material/experiential nature, perceived price, familiarity and attitude.”*

We appreciate the constructive criticisms and feedback received, and were happy that you and both reviewers see the potential of the submitted work. We appreciate, too, your selection of the main questions of the reviewers that are of your concern and thank you for inviting us to submit a revised version of the manuscript, which addresses the points raised during the review process.

We found it interesting that the two reviewers have a very different view about the paper, since it allows us to understand how the general public will receive our paper.

Below, we address Reviewer 1’s concerns, since he/she is the one that is more critical of it, pointing out what he/she understands as weaknesses of the paper.

As suggested by you, we carefully consider the points related to the theoretical contribution of the paper and the lack of a clear theoretical framework and related hypotheses. To this extent, we try to address all the concerns raised by Reviewer 1 in order to make it more clear what we aim with this paper.

We look forward to receiving your response to the new version of our revised manuscript and we thank you for your consideration and support to this work.

Kind regards,

Filipe Loureiro

Teresa Garcia-Marques

Duane Wegener

**Reviewers' comments and Authors responses**

Reviewer #1

**Comment 1**: *In principle, I like and support the idea of studying relevant dimensions for consumer products. But right now, based on the introduction, I do not see any theoretical justification for conducting this study. For example, what are the theoretical gaps the authors are trying to address? This is important to gauge the contribution of their work.*

**Response**: Investigators from several research fields (as exemplified in the paper’s introduction, p.3, lines 11-15) will benefit from having access to extensive normative data on various objective and subjective dimensions related to experimental stimuli. Our aim was to share with the scientific community results from our pre-testing of materials regarding a set of theoretically-relevant dimensions of consumer products (as exemplified by research testing whether, for instance, consumers engage in higher scrutiny of complex products [38, 41], or how the material-experiential distinction is operationalized in a continuum [57], or how product familiarity influences the way consumers process information and make decisions [82,88-93], among others). The selected dimensions are highly relevant for the operationalization of variables that need to be either addressed or controlled for in many studies in several research fields. As such, Reviewer 1 is correct in saying that we are not addressing any particular theoretical gap. Instead, we are addressing an operational gap between theories that relate to dimensions of complexity, quality, experiential nature, etc. and stimuli that researchers could profitably use to study those dimensions.

We understand, with the help of the reviewer, that we were not clear or convincing presenting our goals in the first version of the paper. As such, we added some clarity to the first pages of the paper aiming to clarify our present goal as addressing the operational gap between theories to be tested and the stimuli that might be used to address the conceptual variables addressed by the theories. The conceptual variables are addressed through the presentation of norms concerning six product dimensions: product perceived complexity, quality objectivity, material/experiential nature, perceived price, familiarity and attitude. Although such changes were made across all the introduction, we highlight:

Page 3, lines 15-23:

“Researchers from these fields have the need to control their experimental stimuli in order to guarantee the validity of their operationalizations. Such control can be attained either by extensive pretesting or reliance on general norms published in the literature. Our goal here is to share with the scientific community results of a set of norms for people’s perceptions of several consumer products regarding relevant dimensions, allowing for a faster development of appropriate operationalizations that correspond to conceptual variables addressed by a number of theoretical approaches in the extant literature. We thus aim to address the operational gap between theories to be tested and the stimuli that might be used to address the conceptual variables addressed by the theories.”

Page 4, lines 4-12:

“Our approach in the present work is the same followed in many research areas, wherein norms have been developed to allow researchers to select stimuli according to specific attributes in order to achieve appropriate experimental control (see [26]). The present normative database will support future research that wishes to avoid the interference of confounding factors concerning consumer product dimensions, allowing researchers to select product stimuli in a way that controls for these features.

Besides providing a valuable research resource, these consumer product norms may also offer consumer behavior practitioners (such as marketers and advertisers) insightful information as to how consumers perceive products along relevant dimensions.”

**Comment 2**: *Your introduction introduces too many concepts and can be distracting from the main theme. It would also help to define constructs (albeit briefly) like “complexity”, ‘quality objectivity” etc. As researchers you may be familiar with these, but readers may not be. Further, I would like to know what theoretical gaps, specific to your variables of interest currently remain unaddressed.*

**Response**: Thank you for this comment. Based on it, we made an effort to be clearer with regards to the definition of the six dimensions and their relevance for the literature. Although changes were made across all the introduction, we highlight, with regards to this comment:

Page 4, lines 13-22:

“The selection of the consumer product dimensions for this normative study was tied to relevant research and theory in the consumer literature. For instance, we rely on research suggesting that people’s perceptions of product features, such as perceived product complexity, quality objectivity, and relation to material/experiential purchase goals have a strong impact on their purchase decision-making styles (e.g., [21,22,27 (manuscript in preparation)]). Bellow, we review each of these dimensions regarding their relevance and operationalizations across the existing literature.

We first define what “consumer products” are and then provide an overview of the literature that informs researchers and consumers about the relevant dimensions along which people tend to evaluate these products.”

**Comment 3**: *I think the literature review is very simplistic. Consumer behaviour and product decisions can be nuanced, rather than being based on ‘rational’ versus ‘intuition’ dimension. The research should focus on consumer variables (e.g., individual differences), that drives the perception of product differences.*

*Right now, the research lacks a conceptual model. This is tied to my previous point that understanding perceptions of differences based on consumer related variables will make interesting contribution. Right now, all we know that significant differences exist between products across different dimensions. This is quite intuitive and already known.*

**Response**: In this comment, the reviewer emphasizes, once more, the concern regarding the lack of a theoretical framework to our approach. Although our aim is more methodological than theoretical, we understand the relevance of studying consumer variables (e.g., individual differences) that might lead, or not, to different perceptions of these dimensions across different products. We, too, believe that future work should follow-up on this question (and hence had already raised this point in the discussion section of our manuscript). However, we believe that such research pursuits would benefit from having norms across people along the relevant dimensions. Once those are known, one could conduct research aimed at examining how some consumer characteristic would influence the extent to which differences in one or more product dimension are perceived (or used in product evaluations). The reviewer might conceive of ways that such research could be conducted without starting with target products that normatively differ along the product dimensions of relevance to the consumer characteristics under study, but we believe that such pursuits might progress more smoothly and quickly if the researcher can start with a list of potential products that represent variations along the relevant dimensions (and at least some irrelevant dimensions that the researcher might want to control in order to be able to make claims specific to the dimension or dimensions of interest in the research). We hope that the current descriptions in the manuscript appropriately address the proposed goal of our paper – to provide a first set of norms for people’s perceptions of 150 consumer products – (even if this is not the one required to address the reviewer’s concerns). Instead of differentiating between individuals, we differentiate between consumer products (that could then be used as stimuli in future studies aimed at differentiating among individual consumers).

**Comment 4**: *I think the study should gain from research hypotheses. I am not sure what are the hypotheses that researchers are trying to study. Having a hypothesis followed by appropriate empirical analysis would give me more confidence in the scientific nature of findings.*

**Response**: That approach is one we typically use in our own basic (theory-testing) research. However, as we now clarify in the manuscript, the goal of the current study is descriptive – i.e., to map out differences across products that might be used in future hypothesis-testing studies to either create relevant differences across stimuli or to control for differences that happen to be related to the key dimensions but are not intended to be the focus of the research. As such, this descriptive enterprise is not guided by hypotheses other than assuming that products vary along the dimensions of interest (to researchers and theories in consumer behavior, decision making, and related fields).

Please see page 12, lines 2-5:

“The above literature review demonstrates a number of relevant dimensions along which these products can be evaluated. Based on this evidence, we developed a descriptive study that aims to provide a set of norms for people’s perceptions of a pool of 150 consumer products on each of these dimensions.”

**Comment 5**: *I find some of the constructs (e.g. ‘attitude”) are overly simplified. Measures for attitude are quite well established and consist of multiple items. Given the nature of their products, more items would have been used to capture this construct better.*

*I think the reliability for ‘familiarity’ is low. I would therefore doubt the validity of the findings, as to me, the scale items for this construct do not hold together.*

**Response**: The reviewer’s concern about the validity and reliability of our measures are of great relevance. We acknowledge that our measures used relatively few items, but items that had been extensively used in previous research (as referenced on Table 1) and that corresponded well to the constructs they were intended to measure. In the case of product attitudes, these measurement items represented a number of the semantic differential dimensions routinely used since Osgood, Suci, and Tannenbaum (1957) and corresponding quite well to the evaluation of the product along the valenced dimension (bad-good or disfavor-favor) traditionally defining the attitude construct. All other constructs in our study were equally measured following approaches applied by previous research (as reviewed in the introduction and referenced on Table 1).

We understand and are sensitive to the reviewer’s concern about the measurement of product familiarity. By using as a reliability measure the Cronbach’s alpha, we should recognize that this indicator is highly sensitive to the number of items used to measure a construct. The formula for the Cronbach’s alpha coefficient implies that the greater the number of items present in a scale, the greater its reliability. Nevertheless, and because we agree with the reviewer in the essence of the comment regarding the construct of familiarity, we had already addressed this point in the introduction of our paper as well as in the results section:

Page 10, lines 18-2 (p.11):

“Another dimension closely related to product familiarity is purchase frequency. In fact, research has combined measures of how familiar people are with certain products along with how frequently they buy these products in order to create a product familiarity index (e.g., [84,86]). Despite being associated, the use of both measures as an index of product familiarity might be problematic for some products (e.g., one might be extremely familiar with razor blades and its features but only so often purchase such a product). To that extent, in the present examinations, we measured both product familiarity and purchase frequency and separately present the norms for both dimensions.”

Page 17, lines 21-28:

“The internal consistency of items used to evaluate product familiarity was lower in comparison to the other dimensions, however, suggesting that product familiarity and purchase frequency might reflect different dimensions. The mean value for each of the three items is presented individually for all products in the Supplementary Materials (Tables 1-6), not only for the familiarity/acquisition frequency dimension but also for the five other dimensions. This allows researchers not only to make use of the average of the three items but also to use of the mean values for each item.”

**Comment 6**: I think the respondents have to answer at least 48X3= 144 items to complete their survey. It is quite obvious that there will be fatigue. Can the researchers clarify, what they did to deal with this issue?

**Response**: Like the reviewer, we, too, were concerned about the participants’ potential task fatigue. This is the reason why we took several measures aiming to avoid fatigue, which we address in the manuscript.

Page 12, lines 17-20:

“To prevent task fatigue and demotivation, each participant evaluated 54 products from the total list of 150 products, for one of the six dimensions described. From those 54 products, participants first evaluated a set of 6 calibration products that spanned the range of each of the six dimensions.”

Page 13, lines 20-1 (page 14):

“No time limits were imposed on responses, and participants were told that there were no correct or incorrect answers and that their personal opinion was of particular interest to the researchers.”

Additionally, since products were randomly ordered for each participant, it would also seem that the incidence of responses to a given product falling late in the presented list would be minimized to ensure that any fatigue-induced response tendencies would be equated across products and not represent the majority of responses for any target product.

**Comment 7:** The discussion needs to be more fleshed out in terms of contribution. Right now, I cannot see any theoretical contribution, but a summary of discussion. If I know what authors claim to have discovered, will it improve the research process (e.g., experimental design etc.). Please let the readers know.

**Response**: In line with our response to the reviewer’s first comment, the goal of the work is methodological rather than theoretical. It will be potentially important, though, in that it will fill the important gap between theoretical attention to the dimensions of complexity, experiential nature, positivity (attitude), etc. and operationalizations of those concepts using specific target stimuli in empirical research (or control for dimensions a researcher wishes not to confound certain dimensions of interest). Specifically, the present normative data will allow researchers to overcome methodological limitations when using consumer products, and to select products according to specific attributes and achieve appropriate experimental control.

To this extent, and in addition to the Supplementary Materials that provides descriptive statistics for all products according to each dimension (ordered in descending order), we will also make available a protected spreadsheet that will allow readers to sort all products according to the selected dimension, allowing for a quick examination of the product’s characteristics across all dimension, as exemplified below:

A paragraph was added to the discussion section regarding the availability of the tool described above:

Page 21, lines 13-17:

“To this extent, in addition to the Supplementary Materials providing descriptive statistics for all 150 consumer products for each dimension, a user-friendly spreadsheet is made available (see Data Availability section), that allows researchers to sort (in ascending or descending order) all products for a specific dimension, allowing for a quick examination of the product’s characteristics across all the dimensions.”

**Comment 8**: Please conduct an additional study with relevant product dimensions but this time around with individual difference variables. If possible, run this study in a different culture. I would like to see the results being replicated, and also driven by consumer differences like the authors have posited in their theory.

**Response**: As mentioned, even though we find such possibilities interesting and we believe that the current product norms would be helpful in selecting appropriate stimuli for studies of individual differences in reactions toward or use of the dimensions in product evaluations, these additional research questions go beyond our current goals with this paper. As such, we address them in the discussion section of the manuscript as new questions to be studied in future research.

Reviewer #2:

The paper deals with a very interesting topic, it was well written and it was pleasant to read.

The introduction section well describes all the relevant literature, no important papers are missing. Overall, the research has been well conducted and the design of the experiment is almost flawless. Most importantly, the results provide a very strong evidence that choosing one dimension can create a set of confounding stimuli.

**Response**: We thank the reviewer for a clear understanding of our aims and the relevance of this research for the support of future research.
