# Supplementary material for: Norms for 150 consumer products: Perceived complexity, quality objectivity, material/experiential nature, perceived price, familiarity and attitude
Source: PLoS One. 2020 Sep 21;15(9):e0238848. doi: 10.1371/journal.pone.0238848 (PMC7505470; doi:10.1371/journal.pone.0238848)
Supplement: Attachment — Submitted filename: Response to reviewers.docx [file pone.0238848.s003.docx]

**RESPONSE LETTER**

Filipe Loureiro

William James Center for Research

ISPA - Instituto Universitário

Lisbon, 1149-041

Sunday, July 26^th^, 2020

Dr. Claudio Soregaroli

Academic Editor

PLOS ONE

Dear Dr. Claudio Soregaroli,

We would like to thank you and the reviewers for the constructive comments in the second review round of the paper *“Norms for 150 consumer products: perceived complexity, quality objectivity, material/experiential nature, perceived price, familiarity and attitude.”*

We also thank you for asking the opinion of a third reviewer and for inviting us to submit a revised version of the manuscript addressing the points raised during the second review process.

Below, we address all the concerns raised by Reviewer 1 and 3 and, as suggested by you, we carefully consider the points related to the manuscript’s clarity to the reader, and hope to make the paper’s goals and contribution more clear.

We look forward to receiving your response to the new version of our revised manuscript and we thank you for your consideration and support to this work.

Kind regards,

Filipe Loureiro

Teresa Garcia-Marques

Duane Wegener

**Reviewers' comments and Authors’ responses**

Reviewer #3

**Comment 1:** *The paper tackles an interesting topic and I believe that it can be a relevant contribution to the literature. However, I would suggest some revisions to improve the paper.*

*Although I appreciate the paper, you need to be quite committed to read the whole paper and really get all the details of the study. In the current form the reader needs to go back and forth to understand the design (and hence evaluate the results) of the study.*

*The readability of the introduction could be improved. Moreover, many major details of the study are not introduced here, so the reader needs to read much further before getting a clear picture of the study.*

*Given the wide multi-disciplinary readership of Plos-One, I would suggest extending slightly the introduction to help the reader understand the focus and the relevance of the paper. The first two pages of the manuscript are at points a bit cryptic for a first-time reader. Some suggestions: the authors refer to “theory” (end of page 3) without describing them. In this point you do not need to discuss them, as they are discussed in the following sections but to introduce them. You could just add a few words (it could really be only 1-3 words) to characterise the type of theories you are referring to.*

*Similarly, a sentence could be added to clarify in the first page, what you mean by “share results of a set of norms”. The reader at this point does not have a clear picture of what these norms may be. The term norm has different nuances in different research fields and there are “different types” of norms (e.g., descriptive, injunctive).*

**Response:** We thank the reviewer for the comment and suggestions regarding further improving the introduction. Attending to the point raised concerning the wide multi-disciplinary readership of PlosOne, we added additional information to the introduction to help the reader understand the focus and relevance of the paper. Specifically, and as suggested, we clarify the type of theories we refer to in page 3:

Pages 3-4, lines 21-1:

“This work should contribute to a faster development of appropriate operationalizations that correspond to conceptual variables addressed by a number of theoretical approaches in the extant literature (e.g., theories of consumer perception, choice and behavior).”

We also clarify the type of norms we refer to in this work:

Page 3, lines 17-19:

“Such control can be attained either by extensive pretesting of materials or reliance on general norms published in the literature (i.e., norms for people’s shared perceptions of features specific to particular stimuli).”

Page 3, lines 20-21:

“Our goal here is to share with the scientific community a normative dataset for people’s perceptions of several consumer products regarding relevant dimensions.”

Additional information regarding the importance of these norms can also be consulted on page 4, lines 7-12:

“Our approach in the present work is the same followed in many research areas, wherein norms have been developed to allow researchers to select stimuli according to specific attributes in order to achieve appropriate experimental control (see [26]). The present normative database will support future research efforts to avoid the interference of confounding factors concerning consumer product dimensions, allowing researchers to select product stimuli in a way that controls for these features.”

**Comment 2:** *In section 2 ‘Consumer products as multiple dimensional percepts’ you present an interesting discussion on several product dimensions that are important to evaluate. Before the first paragraph it could be useful to add a figure (or a table if preferred) to highlight the 6 dimensions of your framework to build a conceptual map in the mind of the reader. These are well explained in Table 1, but this is at page 15!*

*That said, the literature review is well done and well readable.*

**Response:** We thank the reviewer for the comment and added a figure to page 5.

**Comment 3:** *Methods are presented in good detail, however some details could be specified further. The authors could list in the text of the paper the 6 calibration products and discuss why they were chosen.*

**Response:** As suggested by the reviewer (besides presenting them in bold at the

Supplementary Materials tables), the calibration products are now presented in Table 1,

on pages 15-16. Additionally, we discuss why and how these were chosen:

Page 13, lines 10-12:

“These calibration items were presented first with the aim of providing participants a sense of the range of the stimuli to be evaluated on that dimension (e.g., [111–113]).”

Page 13, lines 14-17:

“The calibration products for each dimension (presented in Table 1 and bolded in Tables 1-6 of the Supplementary Materials) were selected based on previous research and pre-testing (e.g., perceived complexity: [22,34]; quality objectivity: [22]; material/experiential purchase: [21,114]).”

**Comment 4:** *When firstly discussing about the 150 products the authors could start to introduce what categories of products are included in the study, just to give some information to the readers. In the current form a reader needs to skim forward just to get an idea.*

**Response:** As suggested by the reviewer, when presenting our stimuli, we added some

brief information about the categories of products included in the normative dataset.

Page 13, lines 4-6:

“The full list can be consulted in the Supplementary Materials and includes products such as food, clothing, electronics, household products, experiences and services.”

**Comment 5:** *It is not clear if for the calibration products respondents were asked to answer only to the questions of one dimension or of all 6 dimensions. Please specify this better.*

**Response:** We thank the reviewer for pointing this out. In light of this comment we tried to specify this better.

Page 13, lines 7-10:

“To prevent task fatigue and demotivation, each participant evaluated 54 products from the total list of 150 products for one of the six dimensions described. From those 54 products, participants first evaluated a set of 6 calibration products that spanned the range of the single dimension evaluated.”

**Comment 6:** *In the additional material tables, some products are in bold. I might guess (but it should be clearly stated both in the text and the tables - that should be self-explanatory) that these six products are the calibration ones. However, the bold products are not always the same, leaving me with further confusion. The authors clearly state that “From the total 150 products, the same 6 calibration products were used to start the ratings, leaving a total of 144 non-calibration products.”*

**Response:** The indication that the products in bold in the Supplementary Materials correspond to the calibration products was present in the previous version of the manuscript (previous version: p. 13, lines 12-13: “The calibration products for each dimension (in bold on Tables 1-6 of the Supplementary Materials) were selected based on previous research”). This sentence was adjusted in the current version of the manuscript in order to respond to comment 3:

Page 13, lines 14-17:

“The calibration products for each dimension (presented in Table 1 and bolded in Tables 1-6 of the Supplementary Materials) were selected based on previous research and pre-testing (e.g., perceived complexity: [22,34]; quality objectivity: [22]; material/experiential purchase: [21,114]).”

Additionally, this information was also added, as a note, to the tables in the Supplementary Materials.

The reviewer is correct in observing that the calibration products are not the same, as a product that represented the low end of one dimension, for example, might not also represent the low end of another dimension. The calibration products were chosen to play that role for each given dimension, not across all dimensions at once. The variation in calibration products across the assessed dimensions is now made clear in the current version of the manuscript. To avoid the confusion possibly induced by the footnote on page 13, we made some changes:

Page 13, footnote:

“From the total of 150 products, 6 calibration products were chosen to represent the range of values for that dimension and were used to start the ratings, leaving a total of 144 non-calibration products for each dimension.”

**Comment 7:** *The authors could consider to include in the tables in the supplementary material also a column with the number of observations on which the descriptive statistics were calculated on. This could further highlight the calibration products. Now you can only make some inference on the rough size of the sample looking at the confidence intervals. Moreover, it would be useful to specify that in those tables the mean reported is the mean across the three dimension specific questions of all respondents. The tables as they stand now are not self explanatory.*

**Response:** We thank the reviewer for the suggestion. In line with that, we included in the tables in the Supplementary Materials another column with the number of observation for each product. Additionally, we specify that the mean reported is the mean across the three dimension-specific items.

**Comment 8:** *The authors also do not discuss the sampling method adopted. How were people selected (random, quota sampling, etc.)? How representative are they of North America?*

*Given that the authors discuss only descriptive statistics these are strongly dependent on sample composition. The authors state that the different people were assigned to the different groups randomly. Given the reduced size (i.e., around 20) of the product-dimension specific sub-samples, it would be important to discuss if respondents in these subsamples were well differentiated for socio-demographic variables (age, gender, income, education, where they live, etc,) and representative of the target population or not. Did the authors use quota sampling? And if so on what variables?*

*This is important to establish the inferential value of the results.*

**Response:** Participants were selected form an eligible US population size of 32,842 active participants on Prolific. US citizens are well represented in this platform and correspond to the second best represented country on the platform (after UK, with 40,297 active participants). We do not have information on participants’ socio-demographic variables other than age and gender. Cross-stratification options on the platform are only possible on the following factors: sex, age, ethnicity (Simplified US Census).

While quota sampling would indeed be useful to establish a representative sample of the population of North America, it was not an explicit goal of this work to accomplish such a representativeness, but instead, to study people’s general perceptions of certain stimuli according to specific features (as it is the case with other normative studies). The overall sample of participants in this study is, nevertheless, well distributed across gender, and the median age is close to that of the US general population. Additionally, the confidence intervals reported across all dimensions suggest good uniform reliability.

Nevertheless, we do believe that generalizations should be made with caution and recommend that cross-validation of these norms are performed, as it is the case with other normative datasets (as stated on page 20, lines 17-19).

**Comment 9:** *At page 19 when the authors state: “Next, we computed correlations among the six dimensions across the evaluations of all 150 products using products as the unit of analysis” I think they should specify something like “and the mean values over the product specific respondents as observed feature”, if this is what they have done!*

**Response:** In line with the reviewer’s comment we added the information:

Page 19, lines 15-17:

“Next, we computed correlations among the six dimensions across the evaluations of all 150 products, using products as the unit of analysis (i.e., correlating the mean values for each product’s features).”

**Comment 10:** *Result description and discussion is very concise. The authors do not comment much on the specific results and do not comment at all about the products (products are many, but the authors could discuss some interesting examples) leaving much of the work of thinking about the implications of the study to the reader. It would be useful for practitioners if the authors could discuss more deeply their findings also highlighting the practical implications for applied research that would like to take into account their results. This would further highlight the relevance of the study.*

**Response:** In line with the goals of the paper, we highlight the most important specific results (product-related and dimensions-related) along the three reported tables and presented graphic. Specifically, we start by analyzing the evaluations regarding each dimension across all products (Table 2), before we present a list of the most extreme positive and negatively evaluated products for each dimension (Table 3), emphasizing their overall variation across the range of the dimensions evaluated (also supported by Figure 2). Finally, the analysis of the obtained correlations between the six dimensions across all products (Table 4) highlights the practical implications for applied research and suggests the importance of controlling for these product features, by evidencing several significant correlations among the assessed dimensions, suggesting that these are confounded across products.

As mentioned by the reviewer, the products in this dataset are many, but we do believe that the biggest implications of the obtained results are well explored (and fulfill the goals of this paper), introducing the reader to 1) further explore the individual results regarding the 150 products and 2) be able to select consumer products according to specific attributes and facilitate researchers’ choices aimed at achieving appropriate experimental control.

**Comment 11:** *The paper is written in good English, but it should be re-read carefully to fix a few typos (e.g., missing “to” in “relate to” at page 3; Bellow at page 4; “this dimensions” at page 8). The sentence “Consumer product dimensions that have been the focus of attention on research are first reviewed below and then incorporated into our normative study” at page 5 is a bit awkward.*

**Response:** We thank the reviewer for pointing out these potential typos. We believe that the first of these (on page 3) is phrased correctly (i.e., because it uses “to which participants can easily relate” it would not seem correct to add “to” after “relate” – the added “to” would be redundant with “to which,” and the “to which” phrasing is preferred). The other sentence referenced by the reviewer was changed to:

Page 5, lines 13-14:

“We first review some of the consumer product dimensions that have been the focus of research attention (see Figure 1) before operationalizing them in our normative study.”

In addition to paying attention to these particular sentences, we made further revisions wherever any other typos were detected.

Reviewer #1:

**Comment 1:** *Thank you for the revision and detailed reply to my comments. The theoretical contribution still needs to be highlighted, issue with construct (attitude) could have been addressed with an additional study.*

**Response:** We thank the reviewer for the comment, and hope to have contributed to make this work’s goals (as well as its theoretical contribution) more clear with the changes implemented in this second review round. We don’t believe that, within the purposes of this normative dataset, there is an issue per se with the attitude construct or with how it was operationalized (much in line with our previous response to this point in the last review round). We hence believe that an additional study addressing an alternative operationalization to this construct would not significantly benefit the aims and goals of the current work.
